# Feature Selection Analysis and Robustness to Missing Data for Sensor-Based Prognostic Modeling of Alzheimer’s Disease Progression

**DOI:** 10.3390/s26165109

**Published:** 2026-08-12

**Authors:** Vito Ivano D’Alessandro, Filippo Attivissimo, Tiziana Basileo, Luisa De Palma, Anna Maria Lucia Lanzolla, Attilio Di Nisio, The Alzheimer’s Disease Neuroimaging Initiative

**Affiliations:** Department of Electrical and Information Engineering, Polytechnic University of Bari, Via E. Orabona 4, 70125 Bari, Italy; v.dalessandro4@phd.poliba.it (V.I.D.); filippo.attivissimo@poliba.it (F.A.); basileotiziana@gmail.com (T.B.); luisa.depalma@poliba.it (L.D.P.); attilio.dinisio@poliba.it (A.D.N.)

**Keywords:** Alzheimer’s disease (AD), survival models, features selection, missing data

## Abstract

Feature selection (FS) plays a critical role in sensor-based predictive modeling for Alzheimer’s disease (AD), where heterogeneous clinical and neuroimaging measurements generate high-dimensional data with varying degrees of missingness due to incomplete clinical assessment of patients. Effective dimensionality reduction is essential to improve model interpretability, robustness, and generalization performance in sensor-driven healthcare applications. However, a systematic analysis of the interplay between FS strategies, missing-data handling, and prognostic modeling in sensor-derived AD data remains underexplored. In this study, we present a comprehensive and methodologically rigorous evaluation framework for AD prediction using multimodal data from the Alzheimer’s Disease Neuroimaging Initiative (ADNI) cohort. We jointly investigate multiple FS techniques and prognostic models on imputed datasets, systematically varying the number of top-ranked features. To ensure robustness, a K-fold cross-validation (CV) procedure is adopted and only features consistently selected across folds (intersection-based stability criterion) are retained. These stable feature subsets are subsequently evaluated on a test set. To further assess robustness to incomplete sensor measurements, we conduct a sensitivity analysis by varying the tolerated missingness thresholds for feature inclusion, reflecting realistic scenarios of incomplete clinical data availability. In this phase, XGBoost is employed both as a prognostic model and as an embedded FS method, exploiting its native capability to handle missing values and to provide feature-importance rankings based on predictive contribution. The proposed framework enables a systematic assessment of FS stability, predictive performance, and resilience to missing sensor data. Results provide practical methodological guidelines for the development of reliable and generalizable sensor-driven prognostic models for AD in real-world clinical environments.

## 1. Introduction

Alzheimer’s disease (AD) is a progressive neurodegenerative disorder and the most common cause of dementia worldwide [1]. Clinical and neuroimaging studies often collect a large number of variables relative to the number of subjects, increasing the risk of overfitting and reducing model interpretability. Therefore, feature selection (FS) represents a crucial step in predictive modelling, as it aims to identify a subset of informative variables that maximally contribute to discrimination between disease stages while reducing model complexity and improving generalizability [2,3]. A variety of FS strategies have been proposed and applied in the context of AD, including Minimum Redundancy Maximum Relevance (mRMR), ReliefF and embedded techniques such as Least Absolute Shrinkage and Selection Operator (LASSO) regularization or tree-based importance measures [4,5,6,7,8]. While these approaches have demonstrated promising results, existing studies often differ substantially in their methodological choices, making direct comparisons difficult. As a result, it remains unclear which combinations of FS strategies and predictive models are most robust in real-world clinical settings.

Another critical yet often underexplored aspect concerns the handling of missing data, which is especially prevalent in longitudinal clinical studies and multimodal neuroimaging datasets. Missing values may arise from patient dropout, incomplete examinations, motion artifacts, or variability in acquisition protocols. Inappropriately handling missing data can introduce bias, reduce statistical power, and substantially degrade predictive performance [9]. Common strategies include complete-case analysis, removal of variables exceeding a predefined missingness threshold, and various imputation techniques ranging from simple statistical methods to more advanced approaches such as multiple imputation or random forest-based imputation [10,11]. Importantly, decisions regarding tolerated levels of missingness, that is, determining when a variable should be retained, imputed, or discarded, can strongly affect both the selected feature set and the predictive performance [10]. Despite this, most AD-related machine learning (ML) studies adopt fixed or heuristic thresholds without systematically evaluating their impact. A structured investigation of how different missingness thresholds interact with FS method and prognostic model is therefore essential to improve model reliability and reproducibility.

Additionally, the choice of the ML algorithm plays a pivotal role in determining predictive success. Classical models such as Support Vector Machines, Logistic Regression, Random Forests and Cox model have been widely applied in AD research, often yielding competitive results [12,13,14]. More recently, gradient boosting methods, particularly eXtreme Gradient Boosting (XGBoost) [15,16,17,18], have gained attention due to their strong performance on structured data and their inherent ability to handle missing values by learning optimal default split directions. In scenarios where removing incomplete samples or variables would lead to substantial information loss, such models may offer a practical and effective alternative to explicit imputation strategies.

In this work, we propose a systematic and methodological framework to investigate the interaction between FS strategies and survival models for AD prediction, and how missing data influence model performance. Although these aspects have been investigated separately in previous studies, their combined evaluation within a unified framework remains insufficiently explored in the literature. Specifically, we analyze the effect of varying tolerated missingness thresholds for feature inclusion, compare FS approaches, and evaluate prognostic models both with and without intrinsic capabilities for handling incomplete data. This design enables a structured assessment of how data missingness propagates through the FS stage and affects predictive performance and model stability. Another key contribution of this study is the rigorous and systematic evaluation of FS procedures for robust feature identification, addressing a critical gap in the literature where the FS step is often overlooked or insufficiently explored. Furthermore, we investigate the combined effect of FS strategies and ML models across two clinically relevant transitions, cognitively normal (CN) to mild cognitive impairment (MCI), and MCI to AD, to better understand their impact on predictive performance. All experiments are conducted on the Alzheimer’s Disease Neuroimaging Initiative (ADNI) cohort using a strict separation between training and test sets. Model selection and feature stability are assessed via cross-validation (CV) within the training data, while final performance is evaluated on an independent test split (with different subjects) to ensure an unbiased estimate of generalization. This protocol enables a rigorous analysis of FS consistency and predictive robustness within a controlled yet clinically realistic setting. The structure of the paper is as follows. In Section 2, the dataset utilized in this study with the FS methods and prognostic models is presented. In Section 3, the results are shown. Finally, the findings are discussed in Section 4, and conclusions are drawn while further analyses and results are presented in Supplementary Materials.

## 2. Materials and Methods

This study builds upon a data-driven survival analysis pipeline proposed by the authors [18] to investigate disease progression in the ADNI cohort. The pipeline models time-to-conversion to the next clinical stage using multiple prognostic models and FS strategies. To address event-rate imbalance, the dataset was split into training (90%) and test (10%) subsets using a stratified procedure that preserved the proportion of conversion events in each set. The training subset was further partitioned into five folds using stratified CV to ensure consistent event rates across all folds. Model performance was evaluated at two levels: under 5-fold CV on the training set, to assess robustness and stability across different data partitions, and on the independent test set, to provide an unbiased estimate of generalization performance. The C-Index was adopted as performance metric. Features consistently selected across all folds, defined by their intersection, were retained for final evaluation on the test set.

To further investigate the impact of missing data on model performance, a complementary analysis was conducted using XGBoost 3.2.0 on the non-imputed dataset, exploiting its native capability to handle missing values without explicit imputation. In this analysis, features were additionally filtered by systematically varying the tolerated missingness threshold (e.g., the maximum proportion of missing values allowed per feature), to assess how data missingness affects predictive performance.

### 2.1. Dataset

Data used in the preparation of this article were obtained from the ADNI database (adni.loni.usc.edu). This dataset was launched in 2003 as a public–private partnership, led by Principal Investigator Michael W. Weiner, MD. The primary goal of ADNI has been to test whether serial MRI, PET, other biological markers, and clinical and neuropsychological assessment can be combined to measure the progression of MCI and early AD.

In this study, we employed ADNIMERGE, a curated subset of the ADNI database that includes 60 usable features for survival and disease progression analyses.

Preprocessing included the removal of non-informative variables and administrative metadata, the uniform mapping to *NaN* of heterogeneous encodings of missing or non-quantifiable values, and the removal of rows or columns containing only missing values. Records lacking essential identifiers (RID or VISCODE) were also excluded, with RID representing the unique identifier for each patient and VISCODE indicating the specific time point at which a visit was completed. VISCODE was defined as months since baseline and categorical variables were encoded numerically. Missing data were imputed, in this preprocessing stage, using a *k*-nearest neighbors (KNN) imputation strategy with *k* = 5, as it preserves multivariate relationships among clinical, cognitive, and neuroimaging features and can improve classification performance over simpler statistical imputation strategies [9,19], although performance may vary with model choice and missingness patterns. While alternative imputation strategies are available, in this work we do not investigate the optimal imputation method, but rather we assess the robustness of different modelling and FS techniques under a consistent missing-data handling framework. Therefore, we included both imputed and non-imputed scenarios to investigate how missing-data management choices influence model performance and FS stability. After preprocessing, the dataset comprises 826 CN, 1008 MCI and 413 AD participants with at least one baseline visit. Follow-up duration ranged from 0 to 204 months. Participants had a mean age of 72.90 years (STD = 7.38), a balanced sex distribution, and a high average education level.

To model disease progression along the AD continuum, we considered two different clinical transitions: CN to MCI, and MCI to AD, using only baseline data to reflect a real-world setting. In both analyses, conversion to the next diagnostic stage was defined as event of interest, while non-converters were right-censored at their last follow-up visit. Participants who experienced both transitions (from CN to MCI and subsequently to AD) were included only in the CN to MCI analysis, as their baseline diagnosis was CN. Among 826 CN individuals at baseline, 142 converted to MCI (mean time to conversion: 53.11 ± 36.06 months); in parallel, of 1008 participants with baseline MCI, 378 progressed to AD (mean time to conversion: 31.92 ± 26.86 months). The independent test cohorts included 83 subjects out of 826 for the CN-to-MCI transition, with 14 observed conversion events (16.9% event rate), and 101 out of 1008 subjects for the MCI-to-AD transition, with 38 observed conversion events (37.6% event rate).

### 2.2. Feature Selection and Models

To identify the most informative predictors of disease progression for both clinical transitions, five different FS algorithms were applied to the imputed dataset, using a binary event indicator (conversion/no conversion) as target variable. This choice was motivated by the objective of identifying features associated with disease progression and enabling a consistent comparison among different FS strategies within the proposed framework.

ReliefF evaluates feature relevance by computing weights that distinguish informative from less relevant variables [20,21]; CFS selects subsets of features that are highly predictive of the outcome while minimizing inter-feature redundancy [22]; LASSO applies a regularization penalty to shrink coefficients of less informative predictors, retaining only the most relevant variables [23]; mRMR balances feature relevance against redundancy among already selected features [24]; and SelectKBest ranks features according to statistical metrics, retaining the top-k variables for analysis.

To prevent data leakage, FS was performed exclusively on the training set, and the selected features were subsequently used to train the prognostic models. To evaluate feature stability and model robustness, the number of selected features varied from 1 to 50 in steps of 5, where this step size was chosen as a practical compromise between search resolution and computational cost, allowing a sufficiently detailed characterization of performance trends while avoiding an excessive number of model retraining runs; only features consistently selected across all folds were retained for evaluation on the test set. Within each cross-validation iteration, continuous predictors were standardized to zero mean and unit variance using statistics estimated exclusively from the corresponding training fold, and the same transformation was subsequently applied to the associated validation fold. For the final evaluation on the independent test set, scaling parameters were estimated from the entire training set and then applied unchanged to the test data.

The selected features were used to train five survival models: the Cox Proportional Hazards (Cox PH) model, which is a risk-based model that estimates the risk of the event over time without assuming a specific survival-time distribution [25,26]; the Elastic Net Cox model, which extends Cox PH with a regularization penalty to reduce model complexity and mitigate multicollinearity [27]; the Accelerated Failure Time (AFT) model, assuming a Weibull distribution for survival times, which directly relates covariates to survival time, interpreting them as factors that accelerate or decelerate time to event [28]; the Random Survival Forest (RSF), which estimates survival probabilities by aggregating predictions across multiple decision trees trained on bootstrap samples [25]; and XGBoost, a gradient-boosted ensemble method that additionally serves as an embedded FS method through its intrinsic feature importance rankings, and natively handles missing values by learning optimal default split directions during tree construction, a capability not shared by the other models [29,30]. XGBoost was trained using the survival:cox objective, with censoring encoded through the sign of the survival label (positive survival times for observed conversion events and negative survival times for right-censored subjects). Consequently, feature importance values (importance_type = “gain”) are derived from the reduction in the Cox negative log-likelihood achieved at each split, thereby implicitly accounting for the censoring information incorporated into the training objective.

All survival models were evaluated using their default hyperparameter configurations; therefore, the reported results should be considered as off-the-shelf performance rather than the best performance achievable after model optimization. All experiments were performed on a system running Windows 10 (10.0.26200-SP0), using Python 3.11.9 and the following library versions: NumPy 2.4.4, Pandas 2.3.3, scikit-learn 1.8.0, Scikit-survival 0.25.0, lifelines 0.30.3, and XGBoost 3.2.0.

The selected FS algorithms and survival models were chosen for their widespread use in the biomedical ML literature and their ability to represent different methodological approaches. Although other methods exist, the objective of this study was not to exhaustively benchmark all available techniques, but rather to provide a systematic comparison of representative and commonly adopted approaches within a unified framework.

### 2.3. Impact of Missing Data Handling via XGBoost

As a complementary analysis, we evaluated the non-imputed dataset using XGBoost, which natively handles missing values. In this setting, XGBoost was employed both for FS and survival prediction, allowing comparison with the models trained on the imputed dataset. Features were ranked using XGBoost’s built-in feature importance across the 5 CV folds, and only those consistently selected across all folds were retained for each top-k subset. To systematically investigate the effect of data completeness, multiple column-wise missingness thresholds were applied, retaining only features whose proportion of missing values did not exceed the predefined limit. For each combination of missingness threshold and top-k feature subset, model performance was evaluated under 5-fold CV using the mean C-Index, and the stable feature subset was subsequently used to assess generalization on the test set.

## 3. Results

Here, we present results for the training set using 5-fold CV and test set for the previously mentioned FS strategies survival models, while varying the number of variables employed.

### 3.1. Model Performance on the Validation Set Using 5-Fold CV

#### 3.1.1. Performance on the Validation Set of theImputed Dataset

**Figure 1:**
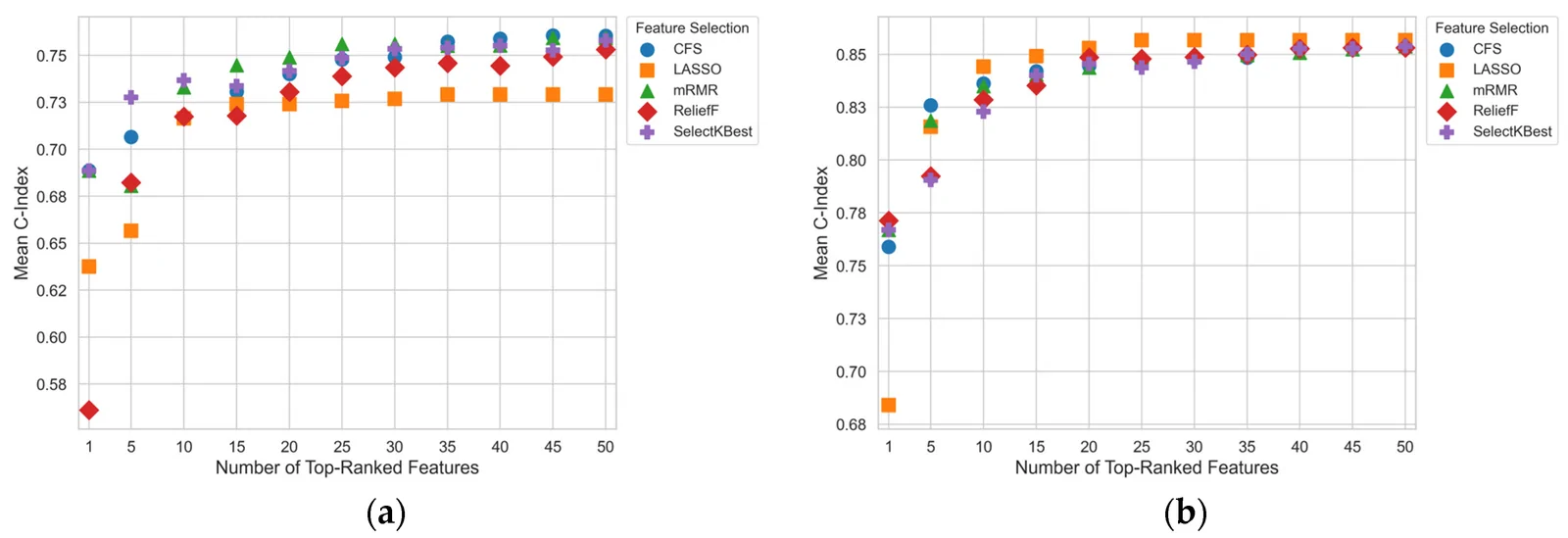
Results of the Cox model on imputed data in terms of mean C-Index of the validation set from 5-fold CV using different FS methods and number of top-ranked features employed for (**a**) the CN-MCI cohort and (**b**) and MCI-AD.

Figure 1 shows Cox model performance on imputed data in terms of mean C-Index on the 5-fold CV using different FS methods. For the sake of brevity, the results for the other survival models are reported in Supplementary Figures S1 and S2. Across all configurations, the mean C-Index displays a plateau beyond approximately 10–15 features, indicating that additional variables did not yield meaningful improvements. This suggests that the most prognostic information is concentrated within a limited feature subset, and that larger models may introduce redundancy without enhancing predictive accuracy.

Moreover, a consistent difference in predictive performance was observed between the two cohorts. Models trained on the CN-MCI transition generally exhibited lower C-Index values than those trained on MCI-AD, likely reflecting the subtler and more heterogeneous clinical changes occurring in the early stages of pathology, which reduce the discriminative power of baseline features. To quantify model stability, the standard deviation (SD) of the C-Index across folds was computed for all configurations and reported in Tables S1 and S2. For CN-MCI, SD values ranged from 0.03 to 0.12, with a mean of approximately 0.064. For MCI-AD, they ranged from 0.01 to 0.18, with a lower mean of approximately 0.022; the upper end of this range was associated with isolated XGBoost configurations. Overall, the later-stage transition therefore showed more stable and consistent predictions, whereas the greater average variability observed for CN-MCI may reflect increased cohort heterogeneity and the higher complexity of the underlying prognostic task.

Regarding the impact of the FS strategy, performance was both cohort- and model-dependent, as shown in Figures S1 and S2. No single FS method consistently outperformed the others across all survival models and number of top-ranked features. For the CN-MCI transition, CFS, mRMR, and SelectKBest generally achieved comparable high validation performance, particularly when combined with the Cox model. For MCI-AD, LASSO produced the highest mean C-Index with Cox, AFT, and ElasticNet, although the differences from the other FS approaches were limited.

#### 3.1.2. Performance on the Validation Set of the Non-Imputed Dataset

In the previous paragraph, we presented the results obtained on the imputed dataset by comparing different prediction models, FS methods, and numbers of features used on the validation set. Building upon these findings, we investigated the impact of missing data by conducting an additional analysis on the original non-imputed dataset. To isolate the effect of the missing-data threshold, this experiment was performed using the XGBoost model together with the corresponding XGBoost-based FS method, while varying the missing-data threshold and reporting the resulting mean C-Index.

**Figure 2:**
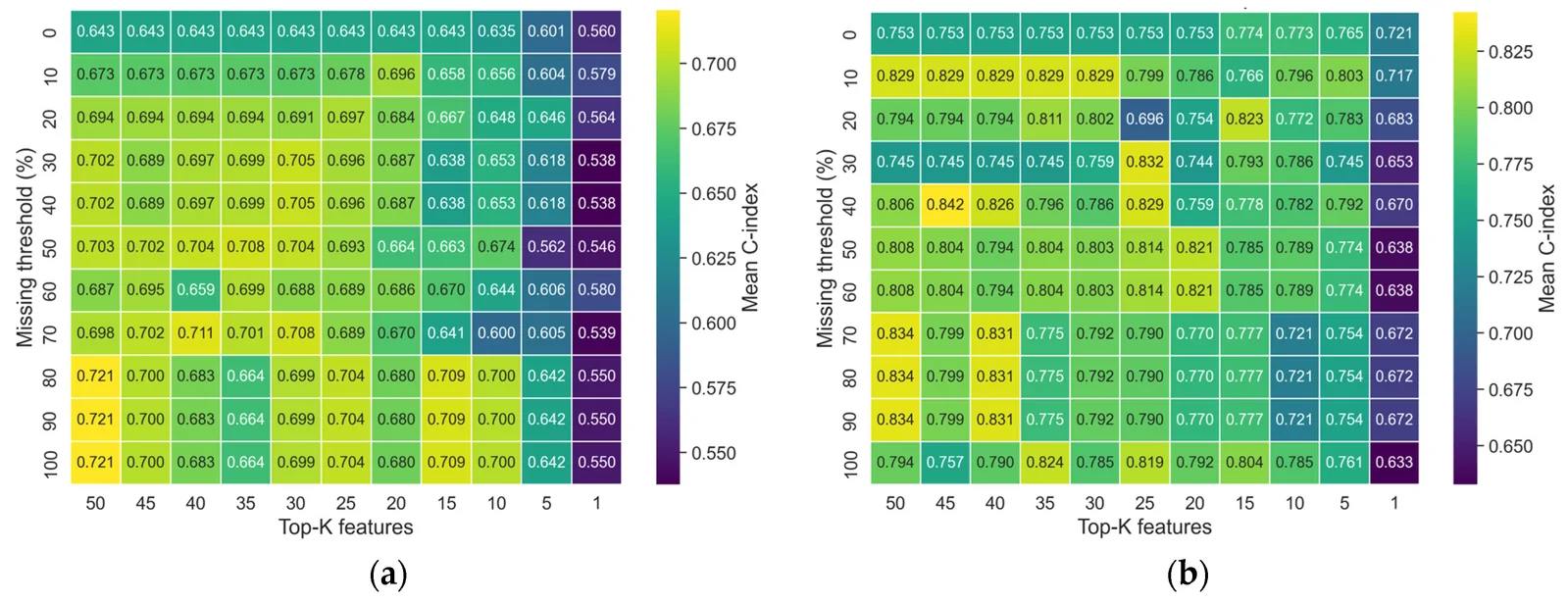
Heatmap of the mean C-Index values on the validation set of non-imputed data using 5-fold CV for (**a**) CN-MCI and (**b**) MCI-AD.

The heatmaps in Figure 2 illustrate how the mean C-Index on the validation set of non-imputed data varies as a function of the number of selected features and the missingness threshold, providing a comprehensive overview of model performance across all tested configurations. The results reveal a clear difference between the two cohorts: the CN-MCI transition shows noticeable performance variability as the missingness threshold increases, indicating higher sensitivity to data incompleteness and feature subset size. In contrast, the MCI-AD cohort exhibits consistently high and stable performance across all configurations, suggesting strong robustness at this disease stage. These findings further corroborate the observation that CN-MCI represents a more challenging prognostic task, whereas MCI-AD appears more predictable.

### 3.2. Feature Selection Stability Analysis

**Figure 3:**
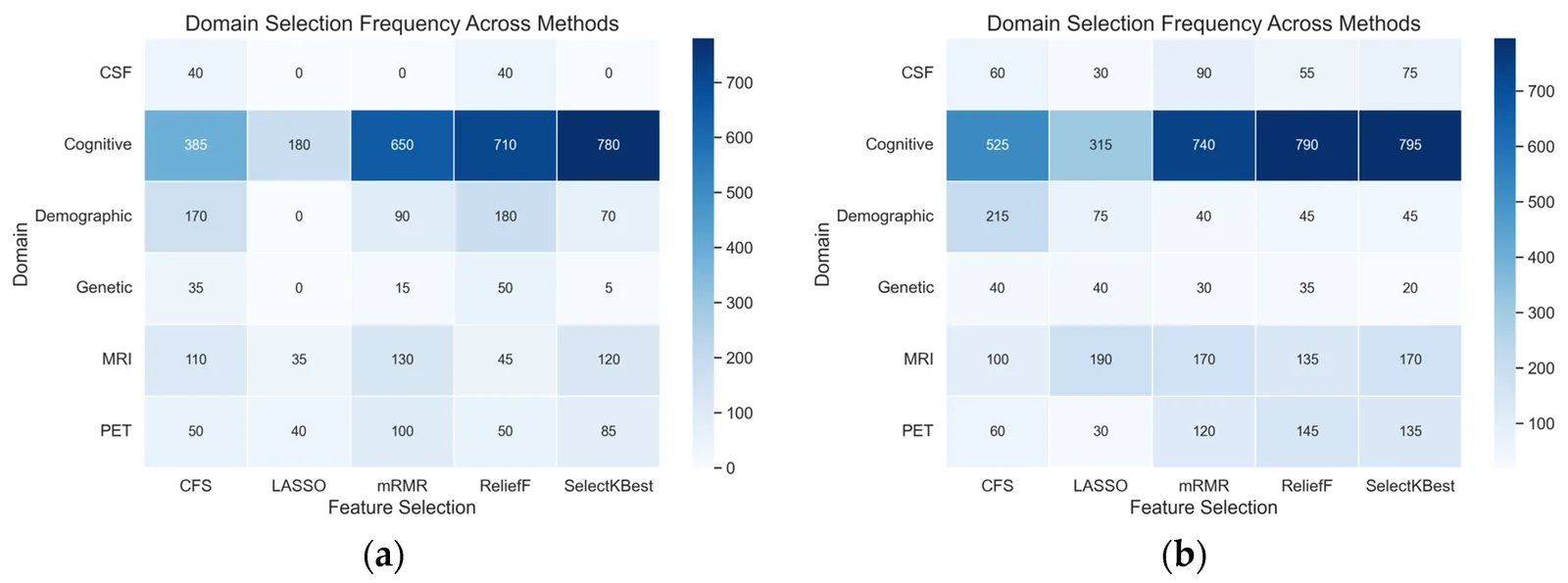
Frequency of the selected feature domains across FS methods, aggregated over the number of top-ranked features, for the (**a**) CN-MCI and (**b**) MCI-AD cohort.

A stability analysis of the selected features across the evaluated FS methods was conducted on the imputed dataset, quantifying the consistency of FS. The results highlight distinct domain-specific selection patterns, as shown in Figure 3. Across different approaches, global cognition and ECog-partner variables consistently emerge as the most robustly selected features across all approaches and both cohorts, whereas demographic and MRI variables proved more method-dependent, being selected only by certain techniques. These findings suggest that the choice of FS method can substantially influence the composition of the final feature subset. The top 15 most frequently selected features for both cohorts are reported in Supplementary Figure S3. Additionally, the same method was applied for the non-imputed data whose results are reported in Figure S7.

### 3.3. Model Performance on the Test Set

#### 3.3.1. Performance on the Test Set of the Imputed Dataset

**Figure 4:**
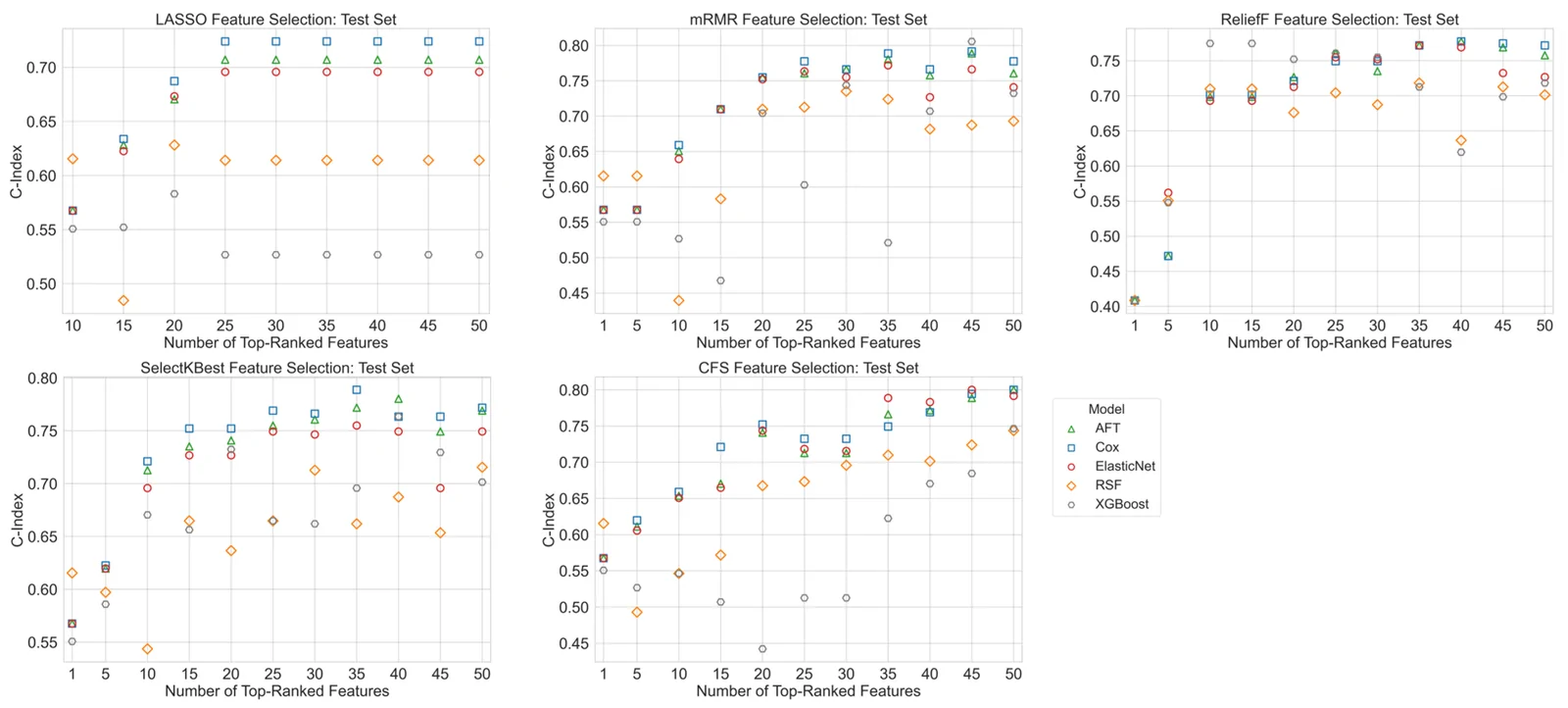
Test set performance for the CN-MCI cohort of the imputed dataset across different FS methods and survival models.

**Figure 5:**
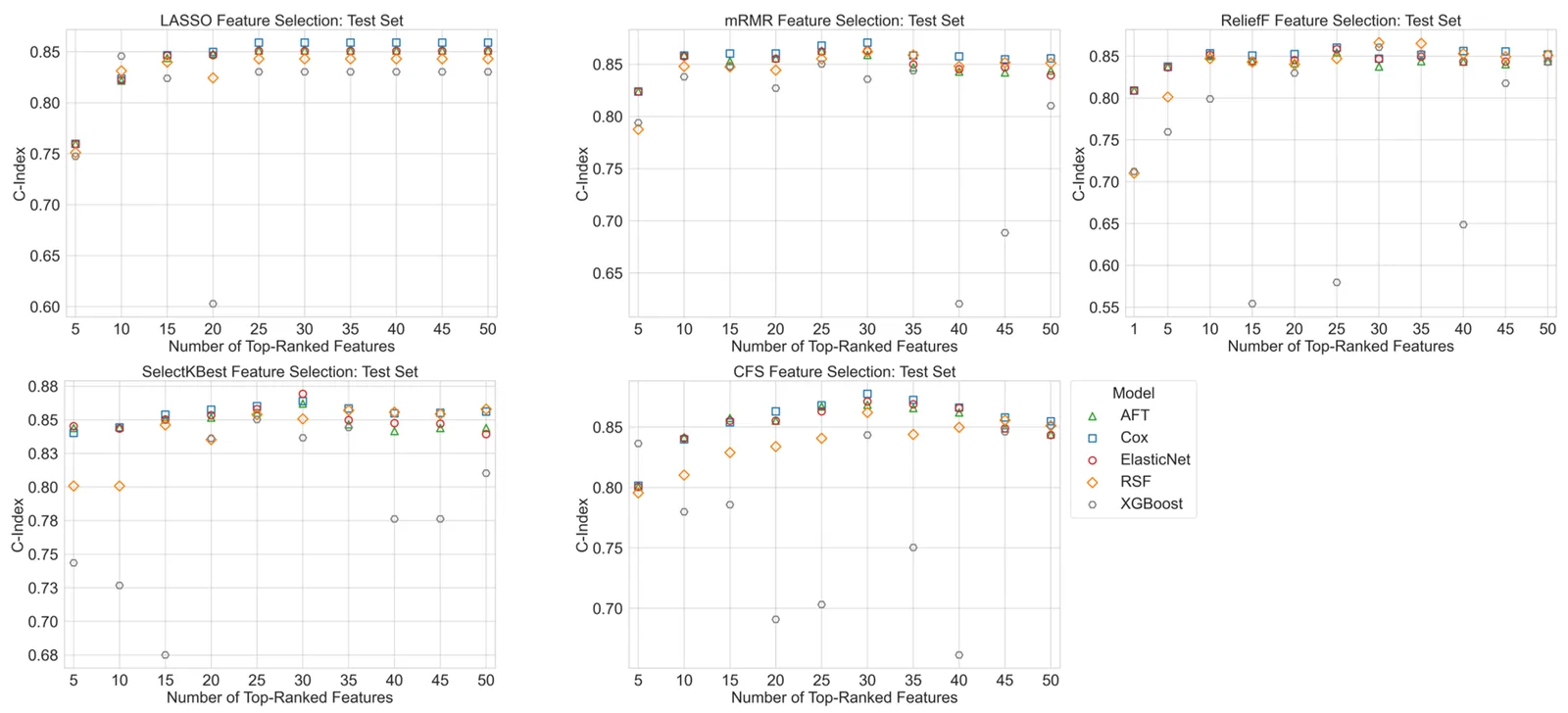
Test set performance for the MCI-AD cohort across different FS methods and survival models.

Figure 4 and Figure 5 show the test set performance using the imputed dataset for the CN-MCI and MCI-AD transitions, respectively. Performance is reported for each combination of FS method, survival model, and number of top-ranked features.

The best configuration associated with each FS method is reported in Table 1. The table includes the number of top-ranked features, the number of features retained in the final model, the C-Index with its corresponding 95% confidence interval, and the computational time required by the entire pipeline. The 95% confidence intervals were estimated using 2000 subject-level percentile bootstrap resamples of the independent test set, without refitting the models.

For the CN-MCI transition, the highest predictive performance was achieved by the mRMR method combined with the XGBoost model, which obtained a C-Index of 0.806 (95% CI: 0.658–0.911) using 37 features. For the MCI-AD transition, the best-performing configuration was obtained using CFS combined with the Cox model, which achieved a C-Index of 0.877 (95% CI: 0.820–0.928) using 22 features.

Although these configurations produced the highest C-Index, the corresponding confidence intervals overlapped substantially with those of the other best-performing configurations. Pairwise comparisons based on 2000 paired subject-level bootstrap resamples confirmed that none of the observed C-Index differences was statistically significant. For CN-MCI, the pairwise differences ranged from 0.006 to 0.082, whereas for MCI-AD they ranged from 0.001 to 0.019. Thus, the independent test results did not provide evidence that any of the leading configurations significantly outperformed the others. Complete results are reported in Supplementary Table S5.

Additionally, two complementary comparisons were conducted to quantify the prognostic contribution of cognitive and imaging variables. The same paired bootstrap procedure described above was used to estimate 95% confidence intervals and two-sided *p*-values for the differences in C-Index between the compared feature sets.

First, the complete selected feature set was compared with the same set after excluding all cognitive and functional variables, in order to determine how strongly model discrimination depended on these assessments. For the CN-MCI transition, excluding cognitive and functional variables reduced the C-Index from 0.806 to 0.465, corresponding to a difference of 0.341 (95% CI: 0.201–0.522; *p* = 0.001). A significant reduction was also observed for MCI-AD, for which the C-Index decreased from 0.877 to 0.749 (ΔC-Index = 0.128, 95% CI: 0.069–0.196; *p* = 0.001). These results indicate that cognitive and functional measurements provide substantial prognostic information for both transitions, with a particularly marked contribution in the earlier CN-MCI stage.

Second, a model based exclusively on the selected cognitive and functional variables was compared with a model combining the same variables with the selected MRI- and PET-derived features. This comparison was performed to assess whether imaging provided additional prognostic information beyond cognitive assessments alone. For CN-MCI, adding imaging features increased the C-Index from 0.623 to 0.783 (ΔC-Index = 0.161, 95% CI: 0.035–0.295; *p* = 0.019), demonstrating a statistically significant incremental contribution. Conversely, for MCI-AD, adding imaging features did not improve discrimination, as the C-Index changed from 0.877 to 0.860 (ΔC-Index = −0.016, 95% CI: −0.047–0.011; *p* = 0.287). Complete results are reported in Supplementary Table S6.

**Figure 6:**
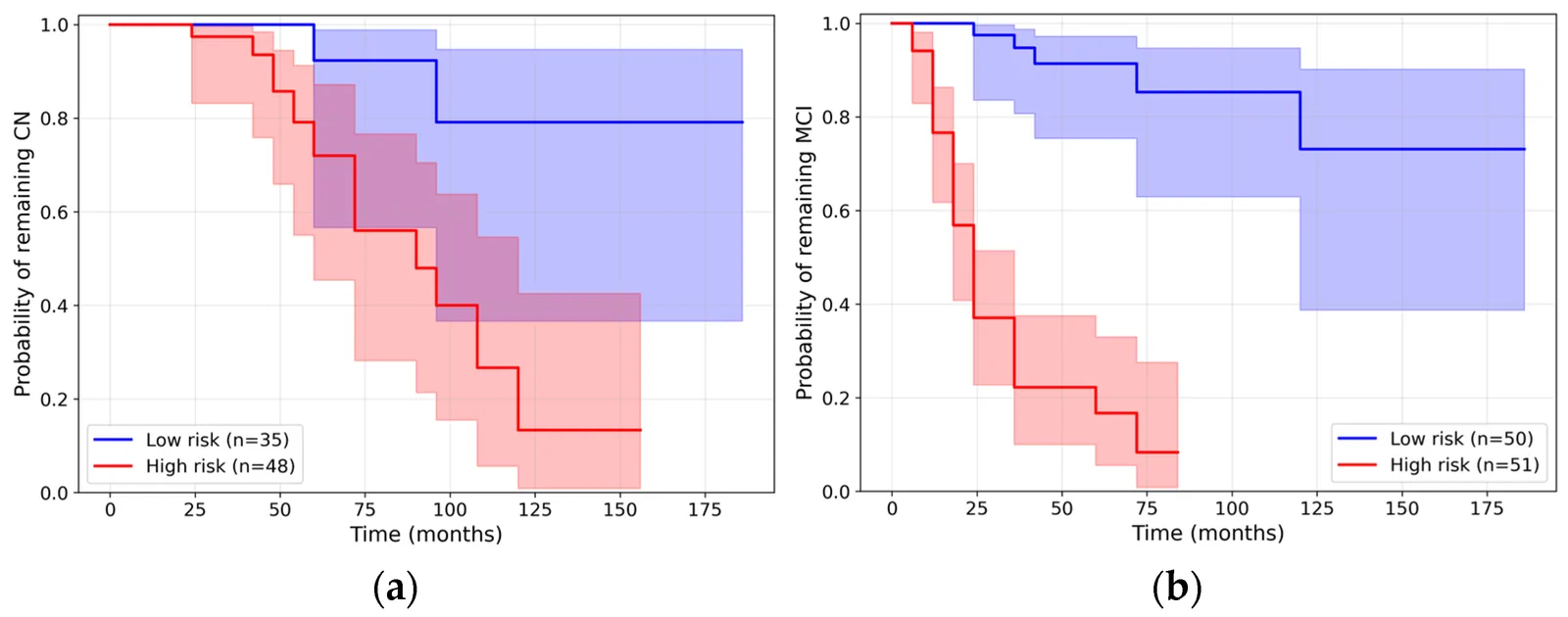
Kaplan–Meier curves for patients stratified into high- and low-risk groups for (**a**) CN-MCI cohort; (**b**) MCI-AD cohort. Patients with higher predicted risk (red) show faster progression compared to lower-risk patients (blue).

To further evaluate the discriminative performance of the best model, we stratified patients into risk groups based on their predictive risk scores, using the median risk score of the training set as cutoff, and we estimated Kaplan–Meier curves, shown in Figure 6.

Both cohorts show a clear and statistically significant separation between the low-risk and high-risk groups, providing strong evidence of the prognostic capability of the proposed models. In the CN-MCI transition, the divergence between the curves is more gradual over time, reflecting a slower and more heterogeneous disease progression, with statistical significance confirmed by the log-rank test (*p* = 0.0031). In contrast, the MCI-AD cohort exhibits an earlier and more pronounced separation between risk groups, with a highly significant log-rank *p*-value (*p* = 2.37 × 10^−12^ ), indicating a stronger and more deterministic progression pattern at this disease stage. Overall, these findings confirm that the best model is able to effectively stratify patients across both transitions, with particularly strong prognostic discrimination in the MCI-AD stage.

To further assess model explainability, SHAP (SHapley Additive exPlanations) values were computed on the test set for the best-performing configuration identified for each transition (Supplementary Figure S4). The analysis showed that the CN-MCI predictions were mainly driven by cognitive performance, amyloid PET burden, and structural brain measures, whereas MCI-AD progression was more strongly characterized by metabolic dysfunction, memory and functional impairment, medial temporal atrophy, and genetic and fluid biomarkers.

Moreover, for each transition, an additional analysis was conducted on the best-performing configuration among those employing Cox as the survival model. More specifically, the estimated coefficients and corresponding hazard ratios are reported in Supplementary Table S3, together with the definitions of the selected features. The proportional-hazards assumption was also assessed for each covariate included in the selected models. None of the covariate-specific tests was statistically significant at the 0.05 level, providing no evidence of violation of the proportional-hazards assumption for either transition (Supplementary Table S4).

#### 3.3.2. Performance on the Test Set of the Non-Imputed Dataset

**Figure 7:**
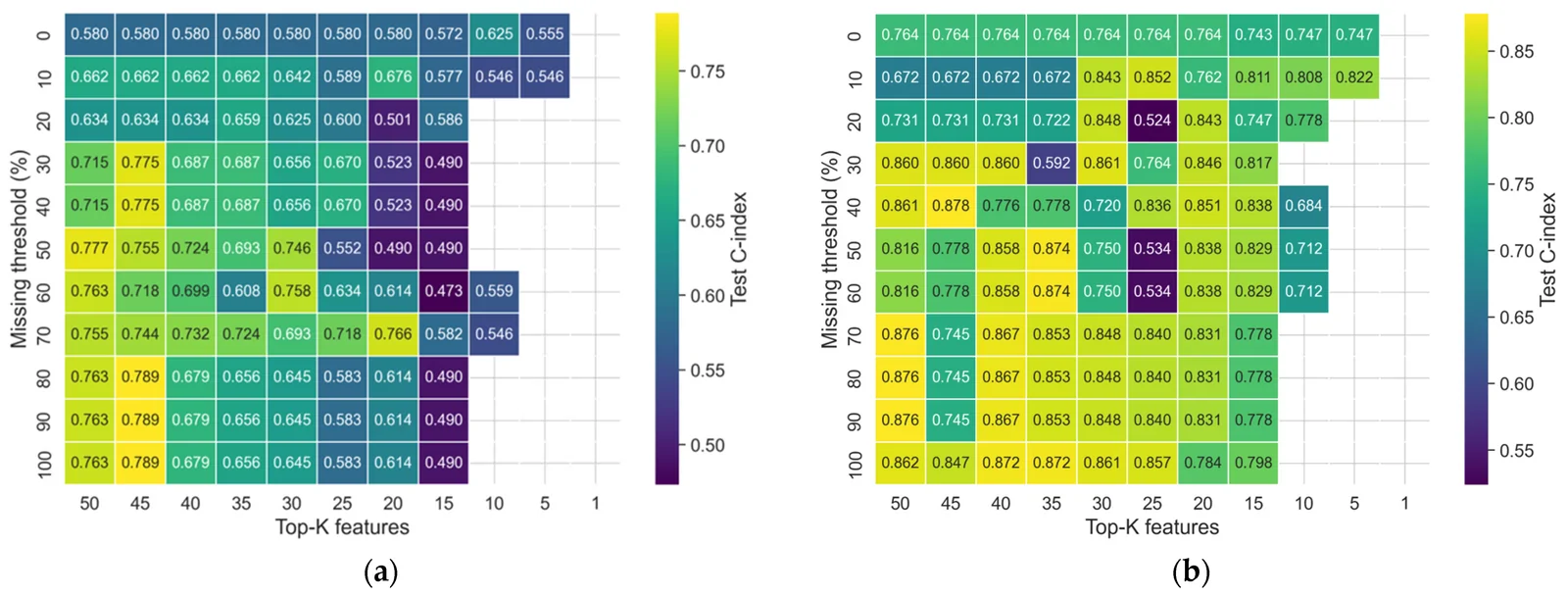
Heatmap of the C-Index values on the test set for (**a**) CN-MCI cohort, (**b**) MCI-AD cohort of non-imputed dataset. Note that the number of overlapping features may vary depending on the tolerated missing-data threshold, for visualization purposes the *x*-axis reports the number of top-k features initially selected in each fold, allowing a fair comparison across different settings.

To complement the analysis conducted on the imputed dataset, model performance was also evaluated using the non-imputed data by varying both the missing-value threshold and the number of top-ranked features. Figure 7 presents the C-Index obtained on the independent test set for each combination of these parameters. The best configuration obtained for the CN-MCI cohort corresponded to 45 top features out of 59 when applying a threshold that retained only columns with 80% of missing values. Among these, 31 features were consistently selected across all 5 CV folds shown in Figure S5, representing the stable feature set and yielding a C-Index of 0.79. Of the 31 features, the first 21 features allow a C-Index of 0.81. For the MCI-AD cohort, the optimal configuration corresponded to 45 top features out of 54 with a missing-value threshold set to 40%. Among these, 41 features were consistently selected across all 5 CV folds (Figure S6), representing the stable feature set. Notably, this subset achieved a C-Index of 0.88, equivalent to that obtained with the top 16 ranked features.

### 3.4. Comparison of XGBoost Performance on Imputed and Non-Imputed Data

To evaluate whether imputation affects model performance, we compared XGBoost results on the test set obtained using imputed and non-imputed data. XGBoost was selected as the survival model for this comparison given its native capability to handle missing values, making it the only model in our framework that can be applied directly to non-imputed data. For the imputed dataset, features were selected using the five FS methods described in Section 2, whereas for the non-imputed dataset, XGBoost itself was employed as the FS method, exploiting its built-in feature importance ranking. Note that for this analysis the missing threshold for non-imputed data is set to 100%, so there is no restriction on the missingness percentage. Table 2 reports the C-Index values on the test set for both cohorts across varying numbers of top-ranked features.

## 4. Discussion

Our analyses of the CN-MCI and MCI-AD cohorts emphasize the interplay between disease stage, FS strategies, and predictive model performance, which is strongly stage-dependent: early CN-MCI transitions benefit from a broader set of features, whereas later MCI-AD progression can be accurately modeled with a smaller feature set. This likely reflects the subtle and heterogeneous nature of cognitive decline in the prodromal phase, which reduces the discriminative power of baseline features and makes risk estimation more challenging.

The combinations yielding the highest observed C-Index differed across cohorts, suggesting that the interaction between FS strategy and survival model may depend on the characteristics of each clinical transition and population. Nevertheless, pairwise comparisons did not demonstrate the statistical superiority of any configuration. Therefore, model selection should also consider differences in computational cost and model complexity, including the number of retained features. For example, in the CN–MCI transition, LASSO combined with Cox provided a substantially more parsimonious and computationally efficient solution than mRMR combined with XGBoost, using 7 rather than 37 features and requiring markedly less execution time (1.28s vs. 14.3s), although its observed C-Index was lower (0.724 vs. 0.806). A similar, but less pronounced, trade-off was observed for MCI–AD transition: LASSO combined with Cox retained 18 features and required 0.87s, compared with 22 features and 4.17 s for CFS combined with Cox, while yielding a lower observed C-Index (0.859 vs. 0.877).

Cognitive variables consistently dominated the most frequently selected features across all FS methods and both cohorts, underscoring their central role in monitoring disease progression along the AD continuum.

A further key finding concerns the stage-specific contribution of the different measurement domains. Excluding cognitive and functional variables significantly reduced discrimination for both CN-MCI (C-Index: 0.806 to 0.465; Δ = 0.341, *p* = 0.001) and MCI-AD (0.877 to 0.749; Δ = 0.128, *p* = 0.001). Additionally, imaging features provided significant incremental value beyond cognitive and functional assessments only for CN-MCI (0.623 to 0.783; Δ = 0.161, *p* = 0.019), whereas no improvement was observed for MCI-AD (0.877 to 0.860; Δ = −0.016, *p* = 0.287). These findings suggest that multimodal imaging measurements provide complementary prognostic information during the earlier transition, when cognitive changes alone may not fully reflect the underlying pathological process. Conversely, after MCI is established, cognitive and functional impairment appears to capture most of the information required for predicting progression to AD.

Although the proposed framework successfully identifies robust and reproducible predictors associated with disease progression, a limitation is that FS was performed using binary conversion status rather than directly incorporating time-to-event information and censoring. This choice enabled a consistent comparison among multiple FS strategies while focusing on the identification of conversion-related predictors. Nevertheless, it may not fully capture variables specifically associated with the timing of conversion. Future extensions of the framework could incorporate survival-aware FS approaches that directly exploit event times and censoring information, further improving the identification of time-dependent prognostic biomarkers. Another limitation is given by the conservative nature of the proposed stability criterion, as features that are informative but not consistently selected across all folds may be excluded. However, this strict intersection-based strategy was adopted to prioritize reproducibility and robustness, reducing the dependence of the selected feature set on individual data partitions.

The analysis on the non-imputed data using XGBoost revealed that there are distinct selected features for distinct transitions. More in-depth, the most frequently selected feature in CN-MCI was mPACCdigit, whereas in MCI-AD was mPACCtrailsB. Additionally, it was revealed that structural brain changes and subtle executive dysfunction are more relevant in early stages, while memory decline and global cognitive impairment become prominent in later disease progression, supporting the hypothesis that distinct biological and cognitive mechanisms dominate different phases of the AD continuum. Moreover, XGBoost on non-imputed data achieved predictive performance comparable to the best imputed-data models, relying on a smaller feature subset. This demonstrates that flexible models capable of natively handling missing values can effectively exploit available information without requiring explicit imputation.

A direct comparison with existing studies is not straightforward due to substantial methodological heterogeneity across the literature, including differences in datasets, cohort selection criteria, preprocessing pipelines, FS strategies, imputation methods, and model choices. The main characteristics of the studies considered for comparison are summarized in Table 3, and the key methodological differences with respect to the present work are discussed below. However, while some studies report slightly higher predictive performance, direct comparisons remain challenging due to differences in cohort composition, preprocessing strategies, feature engineering approaches, validation protocols, and modelling assumptions. Therefore, the reported performance values should be interpreted as contextual references rather than as direct benchmarks, since the lack of standardized evaluation protocols across studies may substantially influence the observed C-Index values. Moreover, many existing studies focus on specific feature subsets or single modelling strategies, without providing a systematic assessment of the combined impact of FS, missing-data influence, and survival model choice across the AD continuum. Abuhantash et al. [31] rely on a relatively broad feature set based mainly on demographic variables, cognitive assessments, and comorbidities, without incorporating imaging or CSF biomarkers. Their preprocessing pipeline relies on Empirical Sampling imputation, and no explicit FS strategy is applied. Several survival models are evaluated, including Cox-based approaches, random survival forests, and deep survival models, with external validation performed on the AIBL cohort, obtaining a C-Index of 0.73. Their best-performing Fast RSF model achieves a C-Index of 0.84 for CN-MCI prediction on the held-out ADNI test set. Despite using 37 features, our model achieves a comparable C-Index of 0.81 on imputed data, with only a marginal decrease of 0.03, and shows stable performance even in more constrained settings (21 features and no imputation), highlighting the robustness of the proposed approach.

Jahani et al. [27] investigate MCI-to-AD conversion prediction using the ADNI cohort and compare traditional statistical survival models with ML approaches, including RSF. Their analysis focuses on a selected subset of MCI subjects with at least four follow-up visits, which may improve longitudinal information availability but also introduces a more restrictive cohort selection. Feature reduction is performed through LASSO, without evaluating alternative FS strategies. Using 14 selected features, their RSF model achieves a C-Index of 0.88. Similarly, Sarica et al. [32] investigate MCI-to-AD conversion using only the ADNI-1 cohort, applying missForest imputation and a RSF model with a primary focus on model explainability. Rather than evaluating alternative FS strategies, their analysis relies on feature importance measures derived from the RSF model to identify the most relevant predictors. Furthermore, the shorter follow-up period results in a different event-censoring distribution, making direct performance comparisons challenging. Under these conditions, they report a C-Index of 0.89 using 43 features. Within our unified benchmarking framework, which systematically compares multiple FS strategies, survival models, and missing-data handling approaches, we achieve a C-Index of 0.88 using 22 features selected by CFS on KNN-imputed data. Notably, when considering 16 non-imputed features, the performance remains stable. Finally, Shigemizu et al. [33] investigate MCI-to-AD conversion using a different dataset based on the NCGG Biobank cohort. Their approach focuses on the integration of multi-omics data, including miR-eQTLs and other molecular biomarkers, to develop a prognosis prediction model rather than evaluating alternative FS or survival modelling strategies. The study is conducted on a smaller cohort with a highly specific molecular feature space, making direct comparisons with clinically oriented frameworks challenging. Under these conditions, they report a C-Index of 0.70 for the MCI-AD task.

Despite the comparable results with the literature, our study lacks an external validation dataset to assess the generalizability of the proposed framework across independent cohorts. However, external validation across publicly available datasets remains challenging due to the lack of standardization among cohorts such as ADNI, OASIS, and NCGG Biobank, which differ in terms of acquisition protocols, available variables, clinical assessments, and preprocessing procedures. Rigorous internal validation procedures, together with an independent held-out test evaluation, were adopted to provide a reliable estimate of model performance within the investigated cohort.

Another limitation of the study is the absence of model hyperparameter tuning. Consequently, the results do not establish an absolute ranking among the models employed, but rather provide evidence on how FS strategies and survival models interact across different disease transitions.

**Table 3:** Literature comparison of Datasets, FS Strategies, and Models in AD Progression Prediction Studies. The results of our work are highlighted in bold.

Study	Test Dataset	Training Dataset	Training Sample Size	Number of Features	FS Method	Imputation Method	Model	Task	C-Index
Shigemizu et al. [33]	NCGG Biobank	NCGG Biobank	98	27	−	IMPUTE2, only for genotype	Cox PH	MCI to AD	0.70
Jahani et al. [27]	ADNIMERGE	ADNIMERGE	631	14	Cox LASSO	missForest	RSF	MCI to AD	0.88
Abuhantash et al. [31]	ADNI	ADNI	494	43	−	Empirical Sampling	Fast Random Forest	CN to MCI	0.84
Sarica et al. [32]	ADNI-1	ADNI-1	309	43	−	missForest	RSF	MCI to AD	0.89
**Our work**	**ADNIMERGE**	**ADNIMERGE**	**743**	**37**	**mRMR**	**KNN**	**XGBOOST**	**CN to MCI**	**0.81**
			**907**	**22**	**CFS**	**KNN**	**COX**	**MCI to AD**	**0.88**
			**743**	**21**	**XGBOOST**	**None**	**XGBOOST**	**CN to MCI**	**0.81**
			**907**	**16**	**XGBOOST**	**None**	**XGBOOST**	**MCI to AD**	**0.88**

## 5. Conclusions

In this study, we presented a systematic and unified framework for evaluating FS strategies, survival models, and missing-data handling in predicting AD progression from multimodal clinical and neuroimaging data. FS stability was assessed through an intersection-based criterion across CV folds, ensuring that only consistently selected features are retained for final evaluation, an aspect frequently overlooked in the existing literature, where features are often chosen a priori without assessing their stability. Moreover, compared with frequency-based stability thresholds, the intersection criterion provides a stricter definition of feature stability by retaining only predictors consistently identified across all data partitions, thereby reducing the influence of sample variability and improving reproducibility. All experiments were conducted under a leakage-free protocol, with FS assessed within the training set and performance evaluated on an independent test set.

Our findings highlight different outcomes. Predictive performance is stage-dependent: CN-MCI transitions generally benefit from a broader feature set, while MCI-AD progression can be accurately modeled with a smaller subset. Furthermore, cognitive measures consistently emerged as the most informative predictors across all FS methods and both cohorts, as demonstrated by the loss of discrimination observed when these variables were excluded, underscoring their central role in monitoring disease progression along the AD continuum. On the contrary, the incremental contribution of imaging was stage-specific. For CN-MCI, adding imaging features to cognitive and functional assessments significantly improved prediction, indicating that early pathological changes are not fully captured by clinical performance alone and that imaging provides complementary information on amyloid burden and structural brain alterations. Conversely, for MCI-AD, the addition of imaging did not improve discrimination beyond cognitive and functional variables.

Among the evaluated configurations, mRMR combined with XGBoost achieved the highest C-Index, whereas CFS combined with Cox obtained the highest performance for MCI-AD. Although no configuration demonstrated statistical superiority, the evaluated approaches differed in feature-set size and computational cost. More parsimonious solutions, such as LASSO combined with Cox, achieved competitive discrimination using fewer predictors and shorter execution times, highlighting the importance of considering trade-offs between feature dimensionality, model performance, and computational efficiency. Regarding missing-data handling, while KNN imputation enabled traditional survival models to reach optimal performance, XGBoost applied directly to non-imputed data achieved comparable results with fewer features, demonstrating the practical advantage of flexible models that can natively leverage incomplete data without preprocessing. This finding is particularly relevant in real-world clinical settings, where data missingness is persistent and imputation pipelines introduce additional complexity and potential bias.

Overall, our study provides practical guidance for developing reliable, interpretable, and efficient prognostic models for AD. Stage-specific modeling, careful selection and stability assessment of features, and appropriate handling of missing data are essential to achieve a good trade-off between computational cost and predictive performance. These insights can inform future precision-medicine approaches, aiding early identification of individuals at risk for cognitive decline and enabling more targeted clinical decision-making.

## Figures and Tables

**Table 1:** Best result for each FS method and model for both transitions on imputed dataset. The C-Index is reported together with its 95% confidence interval. “Top-ranked features” indicates the number of features initially considered within each CV fold, whereas N° indicates the number of features retained in the final model. The best result for each transition is highlighted in bold.

CN-MCI						MCI-AD					
FS	Model	Top-Ranked Features	N°	C-Index	Time (s)	FS	Model	Top-Ranked Features	N°	C-Index	Time (s)
CFS	ElasticNet	45	30	0.800 (0.662–0.895)	9.22	CFS	COX	30	22	**0.877 (0.820–0.928)**	4.17
mRMR	XGBOOST	45	37	**0.806 (0.658–0.911)**	14.3	mRMR	COX	30	26	0.871 (0.815–0.921)	2.61
SelectKBest	COX	35	28	0.789 (0.654–0.898)	0.48	SelectKBest	ELASTICNET	30	24	0.869 (0.814–0.920)	0.2
ReliefF	AFT	40	34	0.777 (0.643–0.872)	40.35	ReliefF	RSF	30	26	0.866 (0.811–0.916)	29.18
LASSO	COX	25	7	0.724 (0.562–0.852)	1.28	LASSO	COX	25	18	0.859 (0.801–0.912)	0.87

**Table 2:** C-Index values on the test set for CN-MCI cohort and for MCI-AD cohort considering XGBoost as survival model and the imputed and non-imputed data, by varying the FS method and the number of top-ranked features. The numbers in parentheses indicate the number of features actually used, defined as the intersection of the features selected across the five CV folds.

		CN-MCI							MCI-AD				
		Imputed					Non-Imputed		Imputed				Non-Imputed
		CFS	LASSO	mRMR	ReliefF	SelectKBest	XGBoost	CFS	LASSO	mRMR	ReliefF	SelectKBest	XGBoost
Top-ranked features	1	0.55 (1)	/	0.55 (1)	0.41 (1)	0.55 (1)	/	/	/	/	0.71 (1)	/	/
	5	0.53 (3)	/	0.55 (1)	0.55 (4)	0.59 (2)	/	0.84 (2)	0.75 (1)	0.79 (1)	0.76 (3)	0.74 (5)	/
	10	0.55 (4)	0.55 (1)	0.53 (3)	0.77 (7)	0.67 (5)	/	0.78 (4)	0.85 (4)	0.84 (7)	0.80 (7)	0.73 (7)	/
	15	0.51 (5)	0.55 (3)	0.47 (6)	0.77 (7)	0.66 (10)	0.49	0.79 (6)	0.82 (9)	0.85 (10)	0.55 (11)	0.67 (13)	0.80
	20	0.44 (9)	0.58 (5)	0.70 (11)	0.75 (9)	0.73 (12)	0.61	0.69 (10)	0.60 (14)	0.83 (15)	0.83 (16)	0.84 (19)	0.78
	25	0.51 (11)	0.53 (7)	0.60 (16)	0.76 (16)	0.66 (17)	0.57	0.70 (15)	0.83 (18)	0.85 (23)	0.58 (22)	0.85 (21)	0.86
	30	0.51 (13)	0.53 (7)	0.74 (20)	0.75 (23)	0.66 (20)	0.64	0.84 (22)	0.83 (18)	0.84 (26)	0.86 (26)	0.84 (24)	0.86
	35	0.62 (19)	0.53 (7)	0.52 (25)	0.71 (29)	0.70 (28)	0.61	0.75 (25)	0.83 (18)	0.84 (33)	0.85 (33)	0.84 (34)	0.87
	40	0.67 (25)	0.53 (7)	0.71 (34)	0.62 (34)	0.76 (34)	0.65	0.66 (32)	0.83 (18)	0.62 (37)	0.65 (37)	0.78 (39)	0.87
	45	0.68 (30)	0.53 (7)	0.81 (37)	0.70 (39)	0.73 (39)	0.79	0.85 (41)	0.83 (18)	0.69 (40)	0.82 (40)	0.78 (41)	0.85
	50	0.75 (38)	0.53 (7)	0.73 (43)	0.72 (46)	0.70 (44)	0.77	0.85 (43)	0.83 (18)	0.81 (46)	0.84 (45)	0.81 (45)	0.86

## Data Availability

Data used in this work were downloaded from the public dataset available at https://adni.loni.usc.edu/ (accessed on 27 February 2025). The code used to perform the analyses presented in this study is available from the corresponding author upon reasonable request.

## Supplementary Materials

The following supporting information can be downloaded at: https://www.mdpi.com/article/10.3390/s26165109/s1, Figure S1: Comparison of features selection algorithms considering the mean C-Index obtained using a 5-fold CV on the validation set using COX (a), RSF (b), AFT (c), ELASTICNET (d), XGBOOST (e) models for the cohort CN-MCI.; Figure S2: Comparison of features selection algorithms considering the mean C-Index obtained using a 5-fold CV on the validation set using COX (a), RSF (b), AFT (c), ELASTICNET (d), XGBOOST (e) models for the cohort MCI-AD.; Table S1: Mean C-Index (computed over 5-fold CV) and corresponding standard deviation (SD) for all numbers of selected features, feature selection methods, and models for the CN-MCI cohort. (R = RSF, C = Cox, A = AFT, E = ElasticNet, X = XGBoost).; Table S2: Mean C-Index (computed over 5-fold CV) and corresponding SD for all numbers of selected features, feature selection methods, and models for the MCI-AD cohort. (R = RSF, C = Cox, A = AFT, E = ElasticNet, X = XGBoost).; Table S3: Features selected in the best-performing Cox model for both CN to MCI and MCI to AD conversions.; Table S4: Proportional-hazards assumption test results for the covariates included in the best-performing Cox models for the CN-MCI and MCI-AD cohorts on the imputed data. The null hypothesis assumes a constant covariate effect over time; *p* > 0.05 indicates that the PH assumption was not rejected.; Figure S3: Top 15 most frequent features for CN-MCI cohort (a) and MCI-AD cohort (b).; Table S5: Pairwise comparisons of test-set C-Index values among the best-performing configurations for the CN-MCI and MCI-AD transitions. Differences were calculated as the C-Index of configuration A minus that of configuration B. The 95% confidence intervals and two-sided *p*-values were estimated using 2,000 paired subject-level bootstrap resamples of the independent test set. Numbers in parentheses indicate the number of features retained after applying the intersection criterion across the five CV folds. No adjustment for multiple comparisons was applied.; Table S6: Prognostic contribution of cognitive and imaging features for the CN-MCI and MCI-AD transitions on the independent test set. C-Index differences, 95% confidence intervals, and two-sided *p*-values were estimated using 2000 paired subject-level percentile bootstrap resamples without model refitting.; Figure S4: SHAP summary plots obtained from the best-performing configuration identified for (a) the CN-MCI transition and (b) the MCI-AD transition.; Figure S5: (a) Feature importance; (b) impact of number of features on model performance for CN-MCI cohort.; Figure S6: (a) Feature importance; (b) impact of number of features on model performance for MCI-AD cohort.; Figure S7: Frequency of selection for intersecting features among different folds, (a) CN-MCI cohort, (b) MCI-AD cohort.; Table S7: Selected features for the CN-MCI and MCI-AD analyses using non-imputed data, along with their definitions. The table includes features that are partially additional with respect to those reported in the previous table and highlights whether each feature was also selected in the analysis conducted on the imputed dataset, enabling a direct comparison between the two settings.
