# Supplementary material for: Feature Selection Analysis and Robustness to Missing Data for Sensor-Based Prognostic Modeling of Alzheimer’s Disease Progression
Source: Sensors (Basel). 2026 Aug 12;26(16):5109. doi: 10.3390/s26165109 (PMC13517737; doi:10.3390/s26165109)
Supplement: Supplementary file 1 [file sensors-26-05109-s001.zip › sensors-4436645-supplementary.pdf]

Supplementary material

The analysis on the validation set using 5-fold CV has allowed the identification of the following important pattern regarding the mean C-Index. It typically reaches a plateau after selecting a moderate number of features, generally around 10-15 variables. In fact, Figure S1 - Comparison of features selection algorithms considering the mean C-Index obtained using a 5-fold CV on the validation set using COX (a), RSF (b), AFT (c), ELASTICNET (d), XGBOOST (e) models for the cohort CN-MCI. and Figure S2 - Comparison of features selection algorithms considering the mean C-Index obtained using a 5-fold CV on the validation set using COX (a), RSF (b), AFT (c), ELASTICNET (d), XGBOOST (e) models for the cohort MCI-AD. show the plateau for all the models and feature selection methods considered for both the cohort on the imputed data, except for XGBoost, where a different trend is visible.

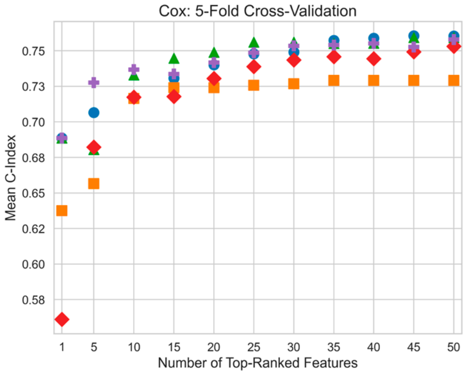

(a)

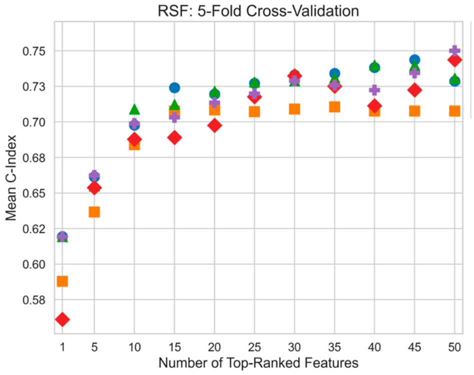

(b)

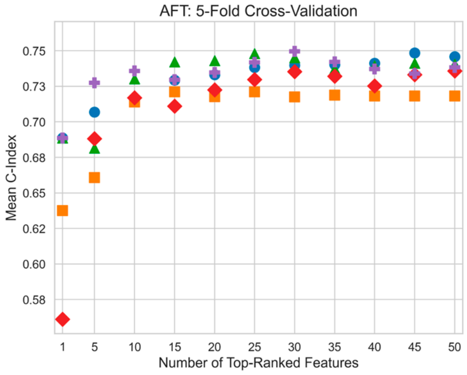

(c)

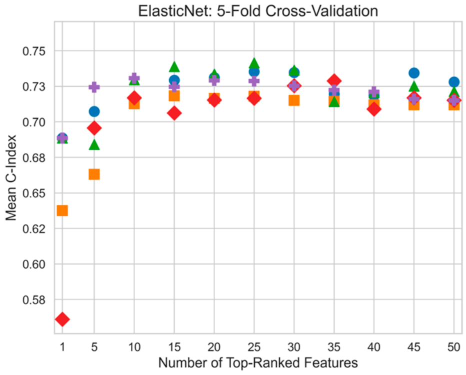

(d)

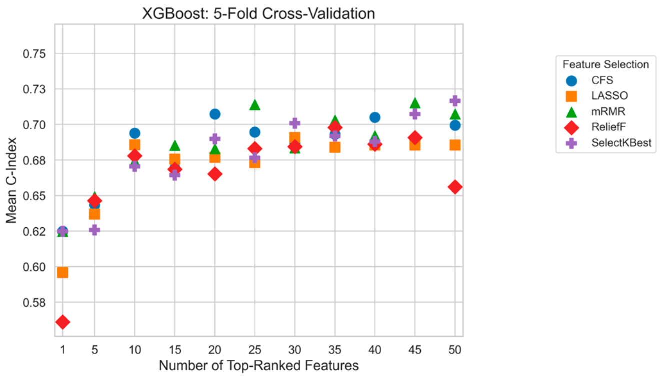

(e)

Figure S1 - Comparison of features selection algorithms considering the mean C-Index obtained using a 5-fold CV on the validation set using COX (a), RSF (b), AFT (c), ELASTICNET (d), XGBOOST (e) models for the cohort CN-MCI.

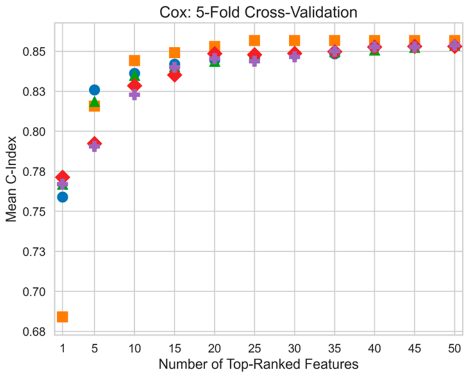

(a)

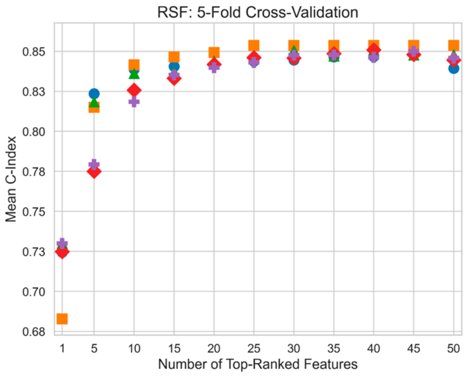

(b)

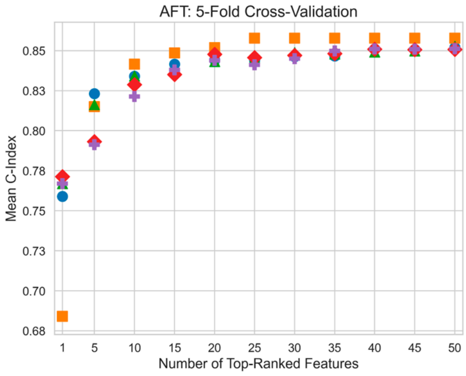

(c)

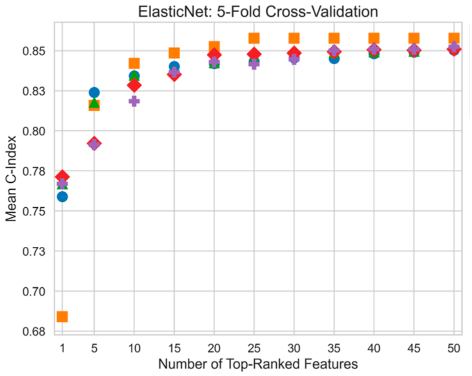

(d)

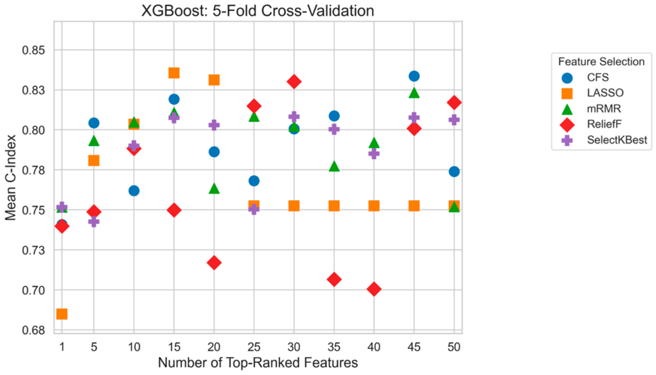

(e)

Figure S2 - Comparison of features selection algorithms considering the mean C-Index obtained using a 5-fold CV on the validation set using COX (a), RSF (b), AFT (c), ELASTICNET (d), XGBOOST (e) models for the cohort MCI-AD.

Table S1**Error! Reference source not found.** reports the Mean C-Index and the corresponding standard deviation (SD) for all numbers of selected features, feature selection methods, and models for the CN-MCI cohort on the imputed data.

Table S1 - Mean C-Index (computed over 5-fold CV) and corresponding standard deviation (SD) for all numbers of selected features, feature selection methods, and models for the CN-MCI cohort. (R = RSF, C = Cox, A = AFT, E = ElasticNet, X = XGBoost).

N	Mean C-Index (SD)																								
	CFS					LASSO					mRMR					ReliefF					SelectKBest				
	R	C	A	E	X	R	C	A	E	X	R	C	A	E	X	R	C	A	E	X	R	C	A	E	X
1	0.62 (0.08)	0.69 (0.07)	0.69 (0.07)	0.69 (0.07)	0.62 (0.07)	0.59 (0.06)	0.64 (0.12)	0.64 (0.12)	0.64 (0.12)	0.60 (0.06)	0.62 (0.08)	0.69 (0.07)	0.69 (0.07)	0.69 (0.07)	0.62 (0.07)	0.56 (0.07)	0.56 (0.07)	0.56 (0.07)	0.56 (0.07)	0.56 (0.07)	0.62 (0.08)	0.69 (0.07)	0.69 (0.07)	0.69 (0.07)	0.62 (0.07)
5	0.66 (0.07)	0.71 (0.06)	0.71 (0.06)	0.71 (0.06)	0.64 (0.07)	0.64 (0.06)	0.66 (0.05)	0.66 (0.05)	0.66 (0.05)	0.64 (0.06)	0.65 (0.07)	0.68 (0.05)	0.68 (0.05)	0.68 (0.05)	0.65 (0.09)	0.65 (0.03)	0.68 (0.06)	0.69 (0.05)	0.70 (0.05)	0.65 (0.03)	0.66 (0.07)	0.73 (0.06)	0.73 (0.06)	0.72 (0.06)	0.63 (0.06)
10	0.70 (0.05)	0.72 (0.06)	0.71 (0.06)	0.71 (0.06)	0.69 (0.08)	0.68 (0.06)	0.72 (0.06)	0.71 (0.06)	0.71 (0.06)	0.69 (0.08)	0.71 (0.05)	0.73 (0.06)	0.73 (0.06)	0.73 (0.06)	0.67 (0.08)	0.69 (0.06)	0.72 (0.06)	0.72 (0.06)	0.72 (0.06)	0.68 (0.07)	0.70 (0.07)	0.74 (0.07)	0.74 (0.07)	0.73 (0.07)	0.67 (0.08)
15	0.72 (0.04)	0.73 (0.05)	0.73 (0.05)	0.73 (0.06)	0.67 (0.06)	0.71 (0.04)	0.72 (0.05)	0.72 (0.05)	0.72 (0.05)	0.68 (0.07)	0.71 (0.04)	0.74 (0.05)	0.74 (0.06)	0.74 (0.06)	0.69 (0.07)	0.69 (0.06)	0.72 (0.06)	0.71 (0.06)	0.71 (0.05)	0.67 (0.09)	0.70 (0.06)	0.73 (0.07)	0.73 (0.07)	0.72 (0.07)	0.66 (0.07)
20	0.72 (0.03)	0.74 (0.04)	0.73 (0.05)	0.73 (0.06)	0.71 (0.03)	0.71 (0.05)	0.72 (0.06)	0.72 (0.06)	0.72 (0.06)	0.68 (0.09)	0.72 (0.05)	0.75 (0.07)	0.74 (0.06)	0.73 (0.07)	0.68 (0.09)	0.70 (0.07)	0.73 (0.06)	0.72 (0.05)	0.72 (0.06)	0.67 (0.09)	0.71 (0.07)	0.74 (0.07)	0.73 (0.06)	0.73 (0.06)	0.69 (0.09)
25	0.73 (0.04)	0.75 (0.06)	0.74 (0.06)	0.74 (0.06)	0.69 (0.06)	0.71 (0.05)	0.73 (0.05)	0.72 (0.05)	0.72 (0.06)	0.67 (0.09)	0.73 (0.05)	0.76 (0.07)	0.75 (0.06)	0.74 (0.07)	0.71 (0.07)	0.72 (0.06)	0.74 (0.06)	0.73 (0.05)	0.72 (0.06)	0.68 (0.08)	0.72 (0.06)	0.75 (0.07)	0.74 (0.07)	0.73 (0.07)	0.68 (0.10)
30	0.73 (0.04)	0.75 (0.05)	0.74 (0.05)	0.73 (0.06)	0.69 (0.06)	0.71 (0.05)	0.73 (0.05)	0.72 (0.06)	0.71 (0.06)	0.69 (0.07)	0.73 (0.05)	0.76 (0.06)	0.75 (0.06)	0.74 (0.06)	0.68 (0.09)	0.73 (0.06)	0.74 (0.06)	0.74 (0.06)	0.73 (0.06)	0.68 (0.09)	0.73 (0.05)	0.75 (0.07)	0.75 (0.06)	0.73 (0.07)	0.70 (0.09)
35	0.73 (0.05)	0.76 (0.06)	0.74 (0.06)	0.72 (0.07)	0.69 (0.09)	0.71 (0.05)	0.73 (0.05)	0.72 (0.06)	0.71 (0.06)	0.68 (0.08)	0.73 (0.06)	0.76 (0.06)	0.74 (0.06)	0.71 (0.08)	0.70 (0.08)	0.73 (0.04)	0.75 (0.06)	0.73 (0.05)	0.73 (0.06)	0.70 (0.07)	0.73 (0.06)	0.75 (0.07)	0.74 (0.07)	0.72 (0.09)	0.69 (0.09)
40	0.74 (0.05)	0.76 (0.07)	0.74 (0.06)	0.72 (0.07)	0.71 (0.07)	0.71 (0.05)	0.73 (0.05)	0.72 (0.06)	0.71 (0.07)	0.69 (0.08)	0.74 (0.05)	0.76 (0.06)	0.74 (0.06)	0.72 (0.07)	0.69 (0.10)	0.71 (0.06)	0.74 (0.06)	0.73 (0.06)	0.71 (0.07)	0.69 (0.08)	0.72 (0.05)	0.76 (0.06)	0.74 (0.07)	0.72 (0.07)	0.69 (0.09)
45	0.74 (0.05)	0.76 (0.06)	0.75 (0.06)	0.73 (0.07)	0.69 (0.08)	0.71 (0.05)	0.73 (0.05)	0.72 (0.06)	0.71 (0.07)	0.69 (0.08)	0.74 (0.05)	0.76 (0.06)	0.74 (0.06)	0.73 (0.07)	0.72 (0.08)	0.72 (0.05)	0.75 (0.06)	0.73 (0.06)	0.72 (0.06)	0.69 (0.10)	0.73 (0.05)	0.75 (0.06)	0.73 (0.06)	0.72 (0.07)	0.71 (0.07)
50	0.73 (0.06)	0.76 (0.06)	0.75 (0.06)	0.73 (0.08)	0.70 (0.07)	0.71 (0.05)	0.73 (0.05)	0.72 (0.06)	0.71 (0.07)	0.69 (0.08)	0.73 (0.06)	0.76 (0.06)	0.74 (0.06)	0.72 (0.07)	0.71 (0.05)	0.74 (0.05)	0.75 (0.06)	0.74 (0.06)	0.72 (0.07)	0.66 (0.12)	0.75 (0.05)	0.76 (0.06)	0.74 (0.06)	0.72 (0.07)	0.72 (0.09)

Table S2 reports the Mean C-Index and the corresponding SD for all numbers of selected features, feature selection methods, and models for the MCI-AD cohort on the imputed data.

Table S2 - Mean C-Index (computed over 5-fold CV) and corresponding SD for all numbers of selected features, feature selection methods, and models for the MCI-AD cohort. (R = RSF, C = Cox, A = AFT, E = ElasticNet, X = XGBoost).

N	Mean C-Index (SD)																								
	CFS					LASSO					mRMR					ReliefF					SelectKBest				
	R	C	A	E	X	R	C	A	E	X	R	C	A	E	X	R	C	A	E	X	R	C	A	E	X
1	0.72 (0.03)	0.76 (0.02)	0.76 (0.02)	0.76 (0.02)	0.74 (0.03)	0.68 (0.06)	0.68 (0.07)	0.68 (0.07)	0.68 (0.07)	0.68 (0.06)	0.73 (0.03)	0.77 (0.01)	0.77 (0.01)	0.77 (0.01)	0.75 (0.02)	0.72 (0.02)	0.77 (0.01)	0.77 (0.01)	0.77 (0.01)	0.74 (0.02)	0.73 (0.03)	0.77 (0.01)	0.77 (0.01)	0.77 (0.01)	0.75 (0.02)

5	0.82 (0.02)	0.83 (0.02)	0.82 (0.02)	0.82 (0.02)	0.80 (0.01)	0.82 (0.02)	0.82 (0.01)	0.82 (0.01)	0.82 (0.01)	0.78 (0.02)	0.82 (0.02)	0.82 (0.02)	0.82 (0.02)	0.82 (0.02)	0.79 (0.02)	0.78 (0.01)	0.79 (0.02)	0.79 (0.02)	0.79 (0.02)	0.75 (0.03)	0.78 (0.01)	0.79 (0.02)	0.79 (0.02)	0.79 (0.01)	0.74 (0.02)
10	0.84 (0.02)	0.84 (0.01)	0.83 (0.02)	0.83 (0.02)	0.76 (0.12)	0.84 (0.01)	0.84 (0.01)	0.84 (0.01)	0.84 (0.01)	0.80 (0.03)	0.84 (0.01)	0.83 (0.01)	0.83 (0.01)	0.83 (0.01)	0.80 (0.04)	0.83 (0.02)	0.83 (0.01)	0.83 (0.01)	0.83 (0.02)	0.79 (0.07)	0.82 (0.02)	0.82 (0.02)	0.82 (0.02)	0.82 (0.02)	0.79 (0.03)
15	0.84 (0.01)	0.84 (0.01)	0.84 (0.01)	0.84 (0.01)	0.82 (0.02)	0.85 (0.01)	0.85 (0.01)	0.85 (0.01)	0.85 (0.01)	0.84 (0.02)	0.84 (0.01)	0.84 (0.02)	0.84 (0.02)	0.84 (0.01)	0.81 (0.03)	0.83 (0.02)	0.84 (0.02)	0.84 (0.02)	0.84 (0.02)	0.75 (0.11)	0.84 (0.01)	0.84 (0.02)	0.84 (0.02)	0.84 (0.02)	0.81 (0.03)
20	0.84 (0.01)	0.84 (0.01)	0.84 (0.01)	0.84 (0.01)	0.79 (0.07)	0.85 (0.01)	0.85 (0.01)	0.85 (0.01)	0.85 (0.01)	0.83 (0.02)	0.84 (0.01)	0.84 (0.01)	0.84 (0.01)	0.84 (0.01)	0.76 (0.07)	0.84 (0.02)	0.85 (0.02)	0.85 (0.01)	0.85 (0.01)	0.72 (0.18)	0.84 (0.01)	0.85 (0.02)	0.84 (0.02)	0.84 (0.01)	0.80 (0.06)
25	0.84 (0.01)	0.85 (0.01)	0.84 (0.01)	0.84 (0.01)	0.77 (0.05)	0.85 (0.01)	0.86 (0.01)	0.86 (0.01)	0.86 (0.01)	0.75 (0.10)	0.85 (0.01)	0.85 (0.01)	0.85 (0.01)	0.84 (0.01)	0.81 (0.03)	0.85 (0.02)	0.85 (0.01)	0.85 (0.01)	0.85 (0.02)	0.81 (0.03)	0.84 (0.01)	0.84 (0.01)	0.84 (0.01)	0.84 (0.01)	0.75 (0.08)
30	0.84 (0.01)	0.85 (0.01)	0.85 (0.01)	0.85 (0.01)	0.80 (0.05)	0.85 (0.01)	0.86 (0.01)	0.86 (0.01)	0.86 (0.01)	0.75 (0.10)	0.85 (0.01)	0.85 (0.01)	0.85 (0.01)	0.85 (0.01)	0.80 (0.05)	0.85 (0.01)	0.85 (0.01)	0.85 (0.01)	0.85 (0.01)	0.83 (0.01)	0.85 (0.01)	0.85 (0.01)	0.85 (0.01)	0.84 (0.01)	0.81 (0.04)
35	0.85 (0.01)	0.85 (0.01)	0.85 (0.01)	0.85 (0.01)	0.81 (0.02)	0.85 (0.01)	0.86 (0.01)	0.86 (0.01)	0.86 (0.01)	0.75 (0.10)	0.85 (0.01)	0.85 (0.01)	0.85 (0.01)	0.85 (0.01)	0.78 (0.07)	0.85 (0.01)	0.85 (0.01)	0.85 (0.01)	0.85 (0.01)	0.71 (0.09)	0.85 (0.01)	0.85 (0.01)	0.85 (0.01)	0.85 (0.01)	0.80 (0.03)
40	0.85 (0.01)	0.85 (0.01)	0.85 (0.01)	0.85 (0.01)	0.75 (0.10)	0.85 (0.01)	0.86 (0.01)	0.86 (0.01)	0.86 (0.01)	0.75 (0.10)	0.85 (0.01)	0.85 (0.01)	0.85 (0.01)	0.85 (0.01)	0.79 (0.04)	0.85 (0.01)	0.85 (0.01)	0.85 (0.01)	0.85 (0.02)	0.70 (0.13)	0.85 (0.01)	0.85 (0.01)	0.85 (0.01)	0.85 (0.02)	0.79 (0.05)
45	0.85 (0.01)	0.85 (0.01)	0.85 (0.01)	0.85 (0.01)	0.83 (0.02)	0.85 (0.01)	0.86 (0.01)	0.86 (0.01)	0.86 (0.01)	0.75 (0.10)	0.85 (0.01)	0.85 (0.01)	0.85 (0.01)	0.85 (0.01)	0.82 (0.01)	0.85 (0.01)	0.85 (0.01)	0.85 (0.01)	0.85 (0.01)	0.80 (0.05)	0.85 (0.01)	0.85 (0.01)	0.85 (0.01)	0.85 (0.01)	0.81 (0.05)
50	0.84 (0.01)	0.85 (0.01)	0.85 (0.01)	0.85 (0.01)	0.77 (0.10)	0.85 (0.01)	0.86 (0.01)	0.86 (0.01)	0.86 (0.01)	0.75 (0.10)	0.85 (0.01)	0.85 (0.01)	0.85 (0.01)	0.85 (0.01)	0.75 (0.04)	0.84 (0.01)	0.85 (0.01)	0.85 (0.01)	0.85 (0.01)	0.82 (0.04)	0.85 (0.02)	0.85 (0.01)	0.85 (0.01)	0.85 (0.01)	0.81 (0.08)

Table S3 reports the features included in the best-performing Cox model for each transition. For each feature, the table provides a brief description, model-estimated beta coefficients and hazard ratios with 95% confidence intervals. The reported parameters were obtained from the models trained on the imputed dataset. A penalized Cox model with L2 regularization (penalizer = 0.1) was used. The regularization shrinks coefficients toward zero, resulting in hazard ratios close to 1 for weak predictors. Confidence intervals were computed but should be interpreted cautiously due to the bias introduced by penalization.

Table S3 - Features selected in the best-performing Cox model for both CN to MCI and MCI to AD conversions.

		CN to MCI				MCI to AD			
**Feature**	**Definition**	**Beta (coef)**	**Hazard Ratio**	**HR 95% CI Lower**	**HR 95% CI Upper**	**Beta (coef)**	**Hazard Ratio**	**HR 95% CI Lower**	**HR 95% CI Upper**
**Biomarkers and Neuroimaging**									
ABETA	Cerebrospinal fluid amyloid-β 42 peptide level.	/	/	/	/	-0.11151	0.89448	0.79299	1.00896
FBB	PET amyloid imaging measure using florbetaben tracer (amyloid load).	0.16259	1.17655	1.02428	1.35145	/	/	/	/
FDG	PET measure of cerebral glucose metabolism.	-0.07004	0.93236	0.81031	1.07278	-0.23503	0.79054	0.70390	0.88786
PTAU	Cerebrospinal fluid phosphorylated tau (p-tau) level.	/	/	/	/	0.10266	1.10812	1.00755	1.21872
Hippocampus	MRI-derived hippocampal volume.	-0.12391	0.88346	0.76058	1.02619	-0.16041	0.85180	0.75820	0.95694
Entorhinal	MRI measure of the entorhinal cortex.	/	/	/	/	-0.16245	0.85006	0.75621	0.95555

		CN to MCI				MCI to AD			
Feature	Definition	Beta (coef)	Hazard Ratio	HR 95% CI Lower	HR 95% CI Upper	Beta (coef)	Hazard Ratio	HR 95% CI Lower	HR 95% CI Upper
MidTemp	MRI measure of middle temporal lobe volume.	-0.09838	0.90631	0.78066	1.05218	/	/	/	/
Ventricles	MRI measure of ventricular volume.	0.08154	1.08495	0.95714	1.22983	/	/	/	/
PIB	PET imaging measure of amyloid deposition using Pittsburgh compound B tracer.	0.02970	1.03014	0.86630	1.22497	/	/	/	/
**Clinical and Cognitive Assessments**									
ADAS11	Alzheimer's Disease Assessment Scale cognitive subscale (11 items).	0.03620	1.03686	0.87684	1.22608	0.12692	1.13533	0.98258	1.31181
ADAS13	Expanded Alzheimer's Disease Assessment Scale cognitive subscale (13 items).	0.07845	1.08161	0.89408	1.30848	0.11221	1.11874	0.94503	1.32440
ADASQ4	Subcomponent (question 4) of the ADAS assessment.	0.08993	1.09409	0.92321	1.29660	0.08790	1.09188	0.94994	1.25503
CDRSB	Clinical Dementia Rating Scale Sum of Boxes; overall dementia severity.	/	/	/	/	0.15195	1.16410	1.04596	1.29559
LDELTOTAL	Logical Memory Delayed Recall total score; verbal memory test.	-0.09829	0.90639	0.76933	1.06786	-0.12634	0.88131	0.77844	0.99778
RAVLT_immediate	Rey Auditory Verbal Learning Test immediate recall (sum of trials).	-0.11995	0.88697	0.74974	1.04931	-0.22189	0.80100	0.69993	0.91666
RAVLT_learning	RAVLT learning score (gain across trials).	-0.10577	0.89963	0.77157	1.04895	0.02816	1.02856	0.91319	1.15852
RAVLT_perc_forgetting	RAVLT percent forgetting; assesses memory retention.	0.09256	1.09698	0.95442	1.26083	/	/	/	/
TRABSCOR	Trail Making Test Part B score; executive function and processing speed.	0.00268	1.00269	0.84816	1.18536	/	/	/	/
FAQ	Functional Activities Questionnaire; everyday functional ability.	/	/	/	/	0.14286	1.15357	1.04301	1.27584
MOCA	Montreal Cognitive Assessment; global cognitive screening.	-0.19542	0.82249	0.69857	0.96839	-0.10734	0.89822	0.79086	1.02015
**Demographic and Genetic**									
AGE	Participant's age in years at assessment.	0.00389	1.00390	0.85335	1.18101	/	/	/	/
APOE4	Apolipoprotein E ε4 allele status.	/	/	/	/	0.18574	1.20411	1.09268	1.32691
PTMARRY=Unknown	Marital status coded as unknown.	/	/	/	/	-0.99776	0.36870	0.06081	2.23541
PTRACCAT=Unknown	Race category unknown.	/	/	/	/	-0.82263	0.43927	0.05596	3.44808
PTRACCAT=Black	Race category coded as Black.	/	/	/	/	-0.27561	0.75911	0.42877	1.34396
PTRACCAT=Hawaiian/Other PI	Race category coded as Hawaiian or other Pacific Islander.	/	/	/	/	-0.24086	0.78595	0.01532	40.31834
**Everyday Cognition (ECog) Scales**									
EcogPtLang	Pt ECog language abilities.	0.02831	1.02872	0.86737	1.22008	/	/	/	/
EcogPtOrgan	Patient (Pt) report on organizational abilities in everyday activities.	0.01469	1.01480	0.85629	1.20266	/	/	/	/
EcogPtPlan	Pt ECog planning/problem solving	0.11752	1.12470	0.95217	1.32851	/	/	/	/
EcogPtTotal	Pt ECog total composite score.	-0.04282	0.95808	0.79442	1.15545	/	/	/	/
EcogSPLang	Study partner (SP) report on language.	0.03465	1.03525	0.87958	1.21848	/	/	/	/
EcogSPDivatt	Study partner report on divided attention.	0.11355	1.12025	0.95379	1.31575	/	/	/	/
EcogSPOrgan	Study partner report on organizational ability.	0.08325	1.08681	0.92159	1.28165	/	/	/	/

		CN to MCI				MCI to AD			
Feature	Definition	Beta (coef)	Hazard Ratio	HR 95% CI Lower	HR 95% CI Upper	Beta (coef)	Hazard Ratio	HR 95% CI Lower	HR 95% CI Upper
EcogSPPlan	Study partner report on planning skills	0.01157	1.01164	0.85442	1.19779	/	/	/	/
EcogSPMem	Study partner report of memory difficulties in everyday functioning.	0.00141	1.00141	0.84046	1.19319	0.18821	1.20709	1.08130	1.34751
EcogSPTotal	Study partner (SP) ECog total composite score.	0.03589	1.03655	0.84706	1.26842	/	/	/	/
**Composite Cognitive Scores**									
mPACCdigit	Modified Preclinical Alzheimer's Cognitive Composite using Digit Symbol Substitution component.	-0.06264	0.93928	0.78057	1.13025	0.02421	1.02451	0.87787	1.19564
mPACCtrailsB	Modified Preclinical Alzheimer's Cognitive Composite using Trail Making Test B component.	-0.07263	0.92995	0.76702	1.12749	-0.11525	0.89115	0.75790	1.04781

Additionally, for these two models, we evaluated the proportional hazard assumptions for each selected feature. As reported in Table S4, none of the covariate-specific tests were significant at the 0.05 significance level for either cohort. Therefore, there was no evidence to reject the PH assumption for any predictor in either model.

Table S4 - Proportional-hazards assumption test results for the covariates included in the best-performing Cox models for the CN-MCI and MCI-AD cohorts on the imputed data. The null hypothesis assumes a constant covariate effect over time; p > 0.05 indicates that the PH assumption was not rejected.

Feature	CN to MCI			MCI to AD		
	Test statistic	p-value	PH assumption	Test statistic	p-value	PH assumption
**Biomarkers and Neuroimaging**						
ABETA	/	/	/	2.88448	0.08944	Not rejected
FBB	0.31087	0.57715	Not rejected	/	/	/
FDG	1.35700	0.24406	Not rejected	0.39123	0.53165	Not rejected
PTAU	/	/	/	2.29899	0.12946	Not rejected
Hippocampus	0.00757	0.93066	Not rejected	0.91012	0.34008	Not rejected
Entorhinal	/	/	/	0.33695	0.56160	Not rejected
MidTemp	0.23226	0.62986	Not rejected	/	/	/
Ventricles	0.01622	0.89867	Not rejected	/	/	/
PIB	0.06032	0.80599	Not rejected	/	/	/
**Clinical and Cognitive Assessments**						
ADAS11	0.00407	0.94913	Not rejected	0.00444	0.94687	Not rejected
ADAS13	0.28971	0.59041	Not rejected	0.10497	0.74594	Not rejected
ADASQ4	0.66908	0.41337	Not rejected	0.39529	0.52953	Not rejected
CDRSB	/	/	/	0.19242	0.66091	Not rejected
LDELTOTAL	0.00012	0.99119	Not rejected	1.17082	0.27923	Not rejected
RAVLT_immediate	0.11039	0.73970	Not rejected	1.48004	0.22377	Not rejected
RAVLT_learning	0.01216	0.91220	Not rejected	0.26860	0.60427	Not rejected
RAVLT_perc_forgetting	0.22532	0.63501	Not rejected	/	/	/
TRABSCOR	0.17998	0.67139	Not rejected	/	/	/
FAQ	/	/	/	1.20041	0.27324	Not rejected
MOCA	0.93820	0.33274	Not rejected	0.29493	0.58708	Not rejected
**Demographic and Genetic**						
AGE	0.31455	0.57490	Not rejected	/	/	/
APOE4	/	/	/	0.33818	0.56088	Not rejected
PTMARRY=Unknown	/	/	/	0.18528	0.66687	Not rejected
PTRACCAT=Unknown	/	/	/	0.05137	0.82070	Not rejected
PTRACCAT=Black	/	/	/	0.65933	0.41680	Not rejected
PTRACCAT=Hawaiian/Other PI	/	/	/	0.00818	0.92792	Not rejected

Feature	CN to MCI			MCI to AD		
	Test statistic	p-value	PH assumption	Test statistic	p-value	PH assumption
**Everyday Cognition (ECog) Scales**						
EcogPtLang	0.22527	0.63505	Not rejected	/	/	/
EcogPtOrgan	0.00510	0.94307	Not rejected	/	/	/
EcogPtPlan	0.03087	0.86052	Not rejected	/	/	/
EcogPtTotal	0.07519	0.78393	Not rejected	/	/	/
EcogSPLang	0.03964	0.84218	Not rejected	/	/	/
EcogSPDivatt	0.12378	0.72497	Not rejected	/	/	/
EcogSPOrgan	2.15853	0.14178	Not rejected	/	/	/
EcogSPPlan	0.47005	0.49297	Not rejected	/	/	/
EcogSPMem	0.05336	0.81731	Not rejected	0.29303	0.58828	Not rejected
EcogSPTotal	0.03581	0.84991	Not rejected	/	/	/
**Composite Cognitive Scores**						
mPACCdigit	0.37069	0.54263	Not rejected	0.31302	0.57583	Not rejected
mPACCtrailsB	0.02896	0.86488	Not rejected	0.09730	0.75510	Not rejected

Figure S3 shows the top 15 most frequent features (on imputed data) for both the cohort. From the analysis of the most frequent features an interesting cohort specific distinction has been highlighted. In the CN-MCI cohort, MOCA is the most frequently selected feature with EcogSPOrgan, RAVLT_immediate, EcogSPLang, Ventricles, and FBB; however, they are not among the top features selected for the MCI-AD cohort. A similar pattern is observed in the MCI-AD cohort: LDELTOTAL is the most frequently selected feature with ADAS13, FAQ, ADASQ4, and CDRSB in this group but they are not selected for the CN-MCI cohort.

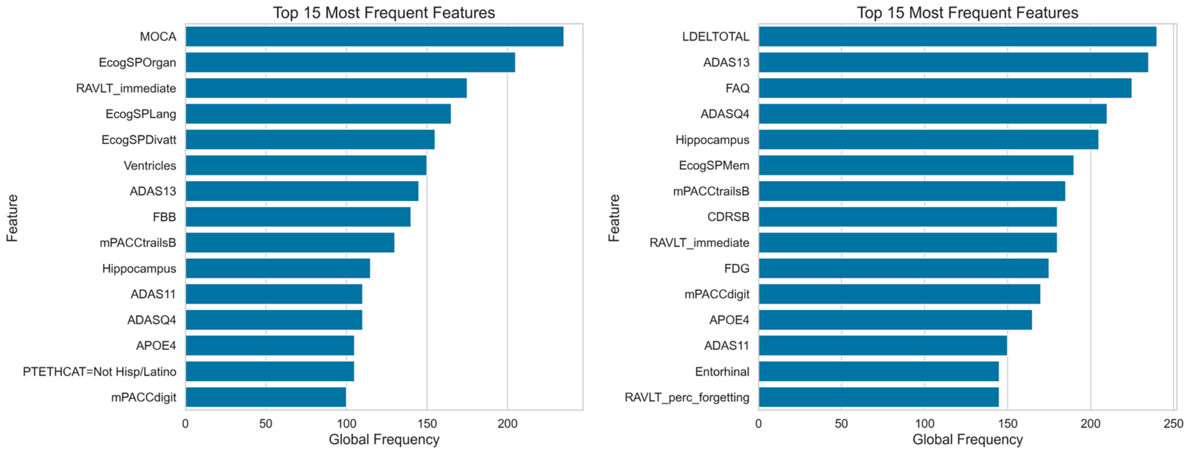

(a) (b)

Figure S3 - Top 15 most frequent features for CN-MCI cohort (a) and MCI-AD cohort (b).

To determine whether the differences in test-set C-Index among the best-performing configurations were statistically significant, a pairwise bootstrap resampling strategy was employed using 2,000 paired subject-level resamples of the independent test set. The results are presented in Table S5. For the CN-MCI transition, the observed differences ranged from 0.006 to 0.082, whereas for the MCI-AD transition they ranged from 0.001 to 0.019. None of the pairwise differences was statistically significant. Therefore, the analysis did not provide evidence that any configuration significantly outperformed the others on the independent test set.

Table S5 - Pairwise comparisons of test-set C-Index values among the best-performing configurations for the CN-MCI and MCI-AD transitions. Differences were calculated as the C-Index of configuration A minus that of configuration B. The 95% confidence intervals and two-sided p-values were estimated using 2,000 paired subject-level bootstrap resamples of the independent test set. Numbers in parentheses indicate the number of features retained after applying the intersection criterion across the five CV folds. No adjustment for multiple comparisons was applied.

**CN-MCI**					**MCI-AD**				
**Configuration A**	**Configuration B**	**ΔC-Index**	**95% CI**	**p-value**	**Configuration A**	**Configuration B**	**ΔC-Index**	**95% CI**	**p-value**
mRMR–XGBoost (37)	CFS–ElasticNet (30)	0.006	−0.110–0.133	0.998	CFS–Cox (22)	mRMR–Cox (26)	0.007	−0.010–0.025	0.467
mRMR–XGBoost (37)	SelectKBest–Cox (28)	0.017	−0.117–0.140	0.755	CFS–Cox (22)	SelectKBest–ElasticNet (24)	0.008	−0.011–0.028	0.418
mRMR–XGBoost (37)	ReliefF–AFT (34)	0.028	−0.081–0.147	0.613	CFS–Cox (22)	ReliefF–RSF (26)	0.011	−0.028–0.051	0.534
mRMR–XGBoost (37)	LASSO–Cox (7)	0.082	−0.040–0.211	0.183	CFS–Cox (22)	LASSO–Cox (18)	0.019	−0.008–0.045	0.170
CFS–ElasticNet (30)	SelectKBest–Cox (28)	0.011	−0.076–0.096	0.779	mRMR–Cox (26)	SelectKBest–ElasticNet (24)	0.001	−0.017–0.020	0.885
CFS–ElasticNet (30)	ReliefF–AFT (34)	0.023	−0.037–0.087	0.409	mRMR–Cox (26)	ReliefF–RSF (26)	0.005	−0.033–0.043	0.774

CFS–ElasticNet (30)	LASSO–Cox (7)	0.076	−0.013–0.197	0.115	mRMR–Cox (26)	LASSO–Cox (18)	0.012	−0.013–0.034	0.298
SelectKBest–Cox (28)	ReliefF–AFT (34)	0.011	−0.070–0.112	0.827	SelectKBest–ElasticNet (24)	ReliefF–RSF (26)	0.003	−0.029–0.035	0.808
SelectKBest–Cox (28)	LASSO–Cox (7)	0.065	−0.020–0.173	0.146	SelectKBest–ElasticNet (24)	LASSO–Cox (18)	0.010	−0.021–0.039	0.456
ReliefF–AFT (34)	LASSO–Cox (7)	0.054	−0.034–0.159	0.283	ReliefF–RSF (26)	LASSO–Cox (18)	0.007	−0.045–0.054	0.777

To assess the domain-specific prognostic contribution of the variables included in the best-performing configuration for each transition on the imputed data, two complementary comparisons were conducted. First, the complete selected feature set was compared with the same set after excluding cognitive and functional variables. Second, the selected cognitive and functional variables were compared with the same variables supplemented by the selected imaging features. The results are reported in Table S6. Exclusion of cognitive and functional variables significantly reduced discrimination in both transitions. The addition of imaging variables provided significant incremental value for CN-MCI, whereas no significant improvement was observed for MCI-AD.

Table S6 - Prognostic contribution of cognitive and imaging features for the CN-MCI and MCI-AD transitions on the independent test set. C-Index differences, 95% confidence intervals, and two-sided p-values were estimated using 2,000 paired subject-level percentile bootstrap resamples without model refitting.

**Transition**	**Comparison**	**Reference feature set (N)**	**Reference C-Index (95% CI)**	**Extended feature set (N)**	**Extended C-Index (95% CI)**	**ΔC-Index (95% CI)**	**p-value**
CN-MCI	Exclusion of cognitive and functional variables	Without cognitive and functional variables (13)	0.465 (0.296–0.612)	All selected features (37)	0.806 (0.658–0.911)	0.341 (0.201–0.522)	0.001
CN-MCI	Addition of MRI- and PET-derived features	Cognitive and functional variables (24)	0.623 (0.484–0.742)	Cognitive and functional variables + imaging features (33)	0.783 (0.643–0.893)	0.161 (0.035–0.295)	0.019
MCI-AD	Exclusion of cognitive and functional variables	Without cognitive and functional variables (10)	0.749 (0.665–0.834)	All selected features (22)	0.877 (0.820–0.928)	0.128 (0.069–0.196)	0.001
MCI-AD	Addition of MRI- and PET-derived features	Cognitive and functional variables (12)	0.877 (0.822–0.925)	Cognitive and functional variables + imaging features (15)	0.860 (0.804–0.911)	−0.016 (−0.047–0.011)	0.287

Figure S4 presents the SHAP summary plots obtained from the best-performing configuration independently identified for each disease transition. For the CN-MCI transition (Figure S4a), mPACCdigit showed the greatest overall influence on model predictions, followed by FBB PET uptake, hippocampal volume, study-partner-reported everyday cognition (EcogSPTotal), ventricular volume, TRABSCOR, and MOCA. Lower mPACCdigit and MOCA scores generally shifted predictions toward a higher progression risk, as did smaller hippocampal and entorhinal volumes. Conversely, higher FBB and AV45 uptake tended to increase the predicted risk, supporting the prognostic contribution of amyloid burden during the earlier disease stage.

For the MCI-AD transition (Figure S4b), FDG PET was the most influential feature, followed by EcogSPMem, RAVLT_immediate, APOE4, entorhinal volume, CDRSB, FAQ, hippocampal volume, and LDELTOTAL. Lower FDG uptake, poorer immediate and delayed memory performance, and reduced entorhinal and hippocampal volumes consistently shifted predictions toward a higher progression risk. Similarly, greater cognitive and functional impairment, reflected by higher EcogSPMem, CDRSB, FAQ, ADAS11, and ADAS13 values, was associated with higher-risk predictions. APOE4 positivity, higher PTAU levels, and lower ABETA concentrations provided additional contributions. Compared with the CN-MCI transition, the MCI-AD configuration showed a greater prominence of metabolic dysfunction, medial temporal atrophy, and cognitive-functional impairment, consistent with a more advanced stage of neurodegeneration.

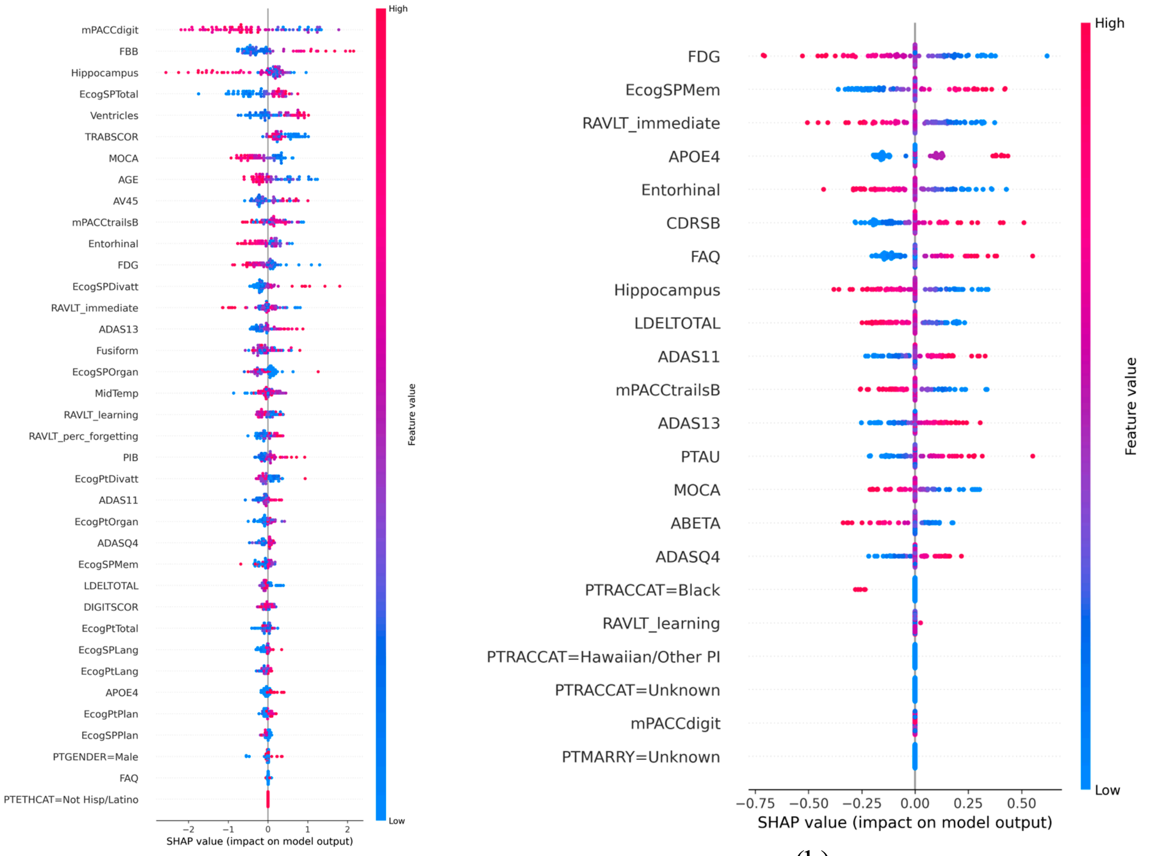

(a) (b)

Figure S4 - SHAP summary plots obtained from the best-performing configuration identified for (a) the CN-MCI transition and (b) the MCI-AD transition.

For the non-imputed CN-MCI cohort, the optimal configuration, considering the C-Index, the number of selected top features, and missing data proportion, corresponded to 45 top features out of 59 when applying a threshold that retained only columns with 80% missing values. Among these, 31 features were consistently selected across all 5 CV folds, representing the stable feature set and yielding a C-Index of 0.79 as shown in Figure S5 - (a) Feature importance; (b) impact of number of features on model performance for CN-MCI cohort.

. As shown, using the first 21 features the model yields a C-Index of 0.81.

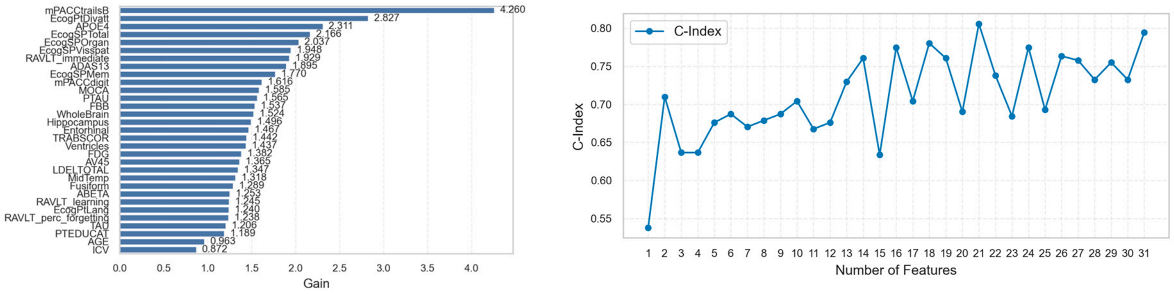

(a) (b)

Figure S5 - (a) Feature importance; (b) impact of number of features on model performance for CN-MCI cohort.

For the MCI-AD cohort, the optimal configuration, considering again the C-Index, the number of selected top features, and proportion of missing data, corresponded to 45 top features out of 54

with a missing-value threshold set to 40%. Among these, 41 features were consistently selected across all 5 CV folds, representing the stable feature set and yielding a C-index of 0.88 as shown in Figure S6 - (a) Feature importance; (b) impact of number of features on model performance for MCI-AD cohort.

. Notably, this subset achieved a C-Index of 0.88, equivalent to that obtained with the first 16 features.

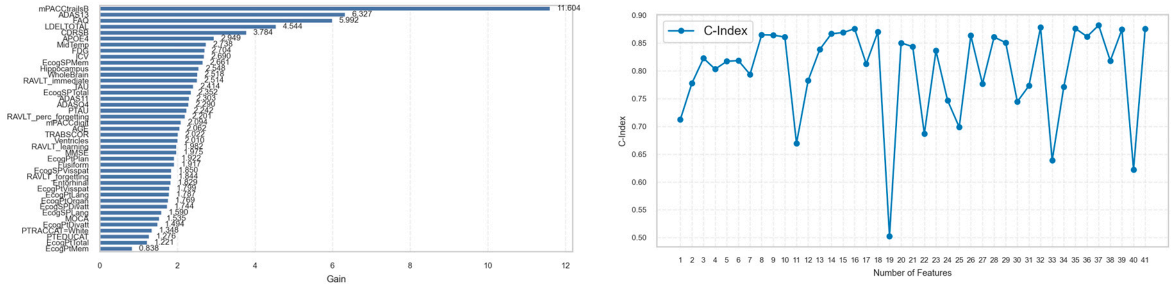

(a) (b)

Figure S6 - (a) Feature importance; (b) impact of number of features on model performance for MCI-AD cohort.

Also, for non-imputed data, a feature stability analysis (Figure S7) was conducted to assess how frequently each feature was selected across different configurations, varying both the number of top-ranked features and the missing-value thresholds.

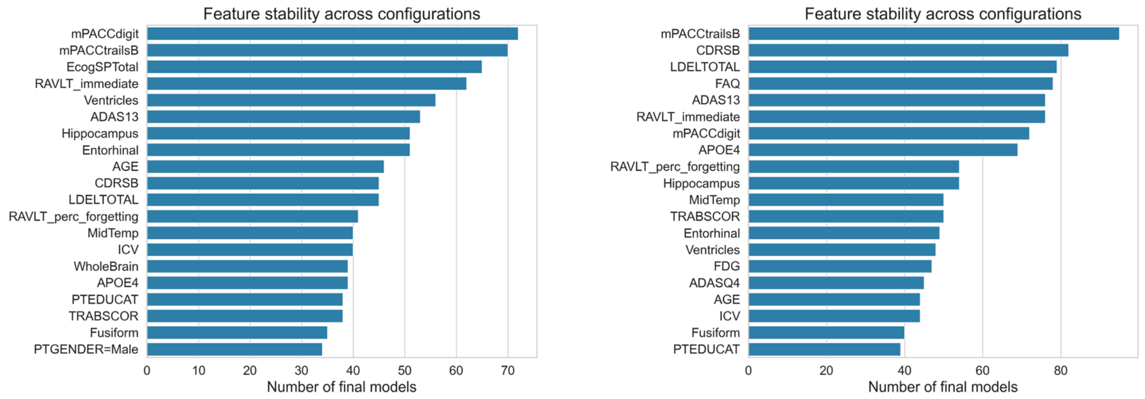

(a) (b)

Figure S7 - Frequency of selection for intersecting features among different folds, (a) CN-MCI cohort, (b) MCI-AD cohort.

Table S7 reports the features selected for the CN-MCI and MCI-AD analyses using non-imputed data, together with their definitions. Compared to the previous Table S3, which presents the features obtained from the imputed dataset, this table includes a partially different set of variables, highlighting features that emerge specifically when no imputation is applied. In addition, it provides a direct comparison by indicating whether each feature was also selected in the imputed-data analysis. This comparison allows us to assess the robustness of the feature selection process and to evaluate the impact of missing data handling on the identification of relevant prognostic variables. This comparison makes it possible to evaluate the overlap between the two approaches, revealing that several of the most frequently selected features are shared across analyses, thereby reinforcing the utility of using non-imputed data.

Table S7 - Selected features for the CN-MCI and MCI-AD analyses using non-imputed data, along with their definitions. The table includes features that are partially additional with respect to those reported in the previous table and

highlights whether each feature was also selected in the analysis conducted on the imputed dataset, enabling a direct comparison between the two settings.

	Definition	Imputed data		Non-imputed data	
		CN-MCI	MCI-AD	CN-MCI	MCI-AD
EcogSPLang	Study partner (SP) report on language.	x			x
EcogSPDivatt	Study partner report on divided attention.	x			x
EcogSPOrgan	Study partner report on organization and planning abilities in daily activities.	x		x	
EcogSPPlan	Study partner report on planning skills.	x			
EcogSPMem	Study partner report on memory abilities in everyday activities.	x	x	x	x
EcogSPVisspat	Study partner report on visuospatial abilities in everyday activities.			x	x
EcogSPTotal	Study partner report on overall cognitive abilities in everyday activities.	x		x	x
EcogPtMem	Pt ECog score for memory.				x
EcogPtVisspat	Pt ECog score for visuospatial abilities.				x
EcogPtPlan	Pt ECog planning/problem solving	x			x
EcogPtOrgan	Pt ECog score for organization.	x			x
EcogPtLang	Pt report on language abilities in everyday activities.	x		x	x
EcogPtDivatt	Pt report on divided attention abilities in everyday activities.	x		x	x
EcogPtTotal	Pt report on overall cognitive abilities in everyday activities.	x			x
FAQ	Functional Activities Questionnaire; everyday functional ability.	x	x		x
CDRSB	Clinical Dementia Rating Scale Sum of Boxes; overall dementia severity.		x		x
MMSE	Mini-Mental State Examination; global cognitive screening test.				x
ADAS11	Alzheimer's Disease Assessment Scale cognitive subscale (11 items).	x	x		x
ADAS13	Expanded Alzheimer's Disease Assessment Scale cognitive subscale (13 items).	x	x	x	x
ADASQ4	Subcomponent (question 4) of the ADAS assessment.	x	x		x
MOCA	Screening test for global cognitive impairment.	x	x	x	x

RAVLT_immediate	Rey Auditory Verbal Learning Test immediate recall (sum of trials).	x	x	x	x
RAVLT_forgetting	Difference between delayed recall and immediate recall (measure of memory retention).			x	x
RAVLT_perc_forgetting	RAVLT percent forgetting; assesses memory retention.	x			x
RAVLT_learning	Measure of verbal learning ability across repeated trials.	x	x	x	x
LDELTOTAL	Logical Memory Delayed Recall total score; verbal memory test.	x	x	x	x
TRABSCOR	Trail Making Test Part B score; executive function and processing speed.	x		x	x
mPACCdigit	Modified Preclinical Alzheimer's Cognitive Composite using Digit Symbol Substitution component.	x	x	x	x
mPACCtrailsB	Modified Preclinical Alzheimer's Cognitive Composite using Trail Making Test B component.	x	x	x	x
ABETA	Cerebrospinal fluid amyloid-β 42 peptide level.		x	x	
PTAU	Cerebrospinal fluid phosphorylated tau (p-tau) level.		x	x	x
TAU	Tau protein levels			x	x
AV45	Amyloid PET imaging measure using the [18F]Florbetapir tracer to assess cerebral amyloid-β plaque burden.	x		x	
FBB	Amyloid PET imaging measure using the [18F]Florbetaben tracer	x		x	
FDG	PET measure of cerebral glucose metabolism.	x	x	x	x
APOE4	Presence of the apolipoprotein E ε4 allele	x	x	x	x
Hippocampus	MRI-derived hippocampal volume.	x	x	x	x
Entorhinal	MRI measure of the entorhinal cortex.	x	x	x	x
MidTemp	MRI measure of middle temporal lobe volume.	x		x	x
Fusiform	MRI measure of the fusiform gyrus volume.	x		x	x
WholeBrain				x	x

	MRI measure of the total brain volume.				
Ventricles	MRI measure of ventricular volume.	x		x	x
ICV	MRI measure of the intracranial volume; used for normalization of brain volumes.			x	x
AGE	Participant’s age in years at assessment.	x		x	x
PTEDUCAT	Years of education completed.			x	x
PTRACCAT=White	Race category white				x
